# Building a Systems Map: Applying Systems Thinking to Unhealthy Commodity Industry Influence on Public Health Policy

**DOI:** 10.34172/ijhpm.2024.7872

**Published:** 2024-04-07

**Authors:** Adam Bertscher, James Nobles, Anna B. Gilmore, Krista Bondy, Amber van den Akker, Sarah Dance, Michael Bloomfield, Mateusz Zatoński

**Affiliations:** ^1^Department of Social and Policy Sciences, Faculty of Humanities & Social Sciences, University of Bath, Bath, UK.; ^2^Centre of Active Lifestyles, Leeds Beckett University, Leeds, UK.; ^3^Department for Health, Faculty of Humanities & Social Sciences, University of Bath, Bath, UK.; ^4^School of Management, Marketing, Business & Society, University of Bath, Bath, UK.; ^5^Department of Psychology, Faculty of Humanities & Social Sciences, University of Bath, Bath, UK.

**Keywords:** Systems Mapping, Complex Adaptive Systems, Participatory Research, Unhealthy Commodities, Commercial Determinants of Health, Non-communicable Diseases

## Abstract

**Background:** Unhealthy commodity industries (UCIs) engage in political practices to influence public health policy, which poses barriers to protecting and promoting public health. Such influence exhibits characteristics of a complex system. Systems thinking would therefore appear to be a useful lens through which to study this phenomenon, potentially deepening our understanding of how UCI influence are interconnected with one another through their underlying political, economic and social structures. As such this study developed a qualitative systems map to depict the complex pathways through which UCIs influence public health policy and how they are interconnected with underlying structures.

**Methods:** Online participatory systems mapping workshops were conducted between November 2021 and February 2022. As a starting point for the workshops, a preliminary systems map was developed based on recent research. Twenty-three online workshops were conducted with 52 geographically diverse stakeholders representing academia, civil society (CS), public office, and global governance organisations (CGO). Analysis of workshop data in NVivo and feedback from participants resulted in a final systems map.

**Results:** The preliminary systems map consisted of 40 elements across six interdependent themes. The final systems map consisted of 64 elements across five interdependent themes, representing key pathways through which UCIs impact health policy-making: (1) direct access to public sector decision-makers; (2) creation of confusion and doubt about policy decisions; (3) corporate prioritisation of commercial profits and growth; (4) industry leveraging the legal and dispute settlement processes; and (5) industry leveraging policy-making, norms, rules, and processes.

**Conclusion:** UCI influence on public health policy is highly complex, involves interlinked practices, and is not reducible to a single point within the system. Instead, pathways to UCI influence emerge from the complex interactions between disparate national and global political, economic and social structures. These pathways provide numerous avenues for UCIs to influence public health policy, which poses challenges to formulating a singular intervention or limited set of interventions capable of effectively countering such influence. Using participatory methods, we made transparent the interconnections that could help identify interventions in future work.

## Background

**Key Messages**

**Implications for policy makers**

- Industry influence on public health policy is not reducible to a single point, but is dispersed throughout a system, and inextricably linked to different political practices and underlying structures.
- The interdependent and complex pathways through which industries influence public health policy suggest that they can adapt to changes in the system, limiting intervention effectiveness over time.
- Strategies to address industry influence on public health policy should cohere with each other and aim to tackle the underlying political, economic and social structures that give rise to unhealthy commodity industry (UCI) influence.
- If the structures underlying UCI influence are not addressed, industry influence may continue despite the implementation of well-intended interventions.

**Implications for the public**

Industries that produce and sell tobacco, alcohol, and unhealthy foods engage in political practices designed to influence public health policy-making, leading to industries remaining insufficiently regulated and policy that is made in industries’ favour. Interventions to address such influence would likely help safeguard public health policy-making and improve its effectiveness in protecting and promoting public health. Interventions should not only be aimed at policy-making, such as accountability and lobbying measures, but should also be aimed at the underlying political, economic and social structures that enable unhealthy commodity industries (UCIs) to exert power and influence. These structures may include privatisation, international trade and investment, and norms on multistakeholder governance. Public health professionals and civil society (CS) actors should support interventions that both improve policy-making processes and aim to change the wider system that drives the accumulation of UCI power. If not, interventions may be ineffective at addressing industry influence on public health policy.

The political practices used by unhealthy commodity industries (UCIs) pose a significant barrier to advancing public health policy and goals. Recent research on the commercial determinants of health (CDoH) has outlined how corporate political practices help shape the policies, policy environments, and underlying political, economic and social structures that drive unhealthy commodity consumption (eg, tobacco, alcohol, and ultra-processed foods), ultimately leading to poor health outcomes and widespread health inequalities.^1^ Similarly, a recent evidence-based taxonomy shows how different UCIs use analogous corporate political practices to influence public health policy at various levels of governance across the world,^2^ which is supported with an increasingly extensive and growing volume of literature.^3-35^ Scholars have suggested that UCI influence is a complex problem, derived from a complex system, and have called for systems thinking approaches to be applied to it.^1,3,36,37^ Systems thinking may therefore help to deepen our understanding of this problem and identify the key solutions. However, to date, no study has explicitly applied systems thinking methods this phenomenon. To answer this call, we aimed to apply systems thinking to map out the complexity and pathways through which UCIs influence public health policy.

 Systems thinking is an approach to studying complex systems,^38-44^ defined as a composition of many interconnected and interdependent elements that function together as a whole.^38,44-47^ These elements interact with one another in such a way that their combined behaviour produces emergent properties and patterns.^41,44,46,48-51^ Systems thinking views phenomena as more than just the sum of its parts; it emphasises understanding the relationships, feedback loops, and dynamic behaviour of a system as a whole.^41,46,48,49,51^ Complex systems are characterised by adaptivity and unpredictability of how a system reacts to change.^44,45,52,53^ They are also characterised by the heterogeneity and interactivity between stakeholders, processes and structures that produce results that may otherwise not exist if these things functioned independently of each other.^44,53,54^

 One key characteristic of a complex system is that it can adapt to change and there is some evidence that industry actors do so. One example is how the tobacco industry adapted to the introduction of the World Health Organization (WHO) Framework Convention on Tobacco Control (FCTC). The WHO FCTC’s Article 5.3 restricted the tobacco industry’s access to policy-makers and, alongside the tobacco industry’s growing denormalisation, reduced its ability to influence policy.^55-57^ In response to this denormalisation, the tobacco industry adapted by investing heavily in corporate social responsibility practices and later increased the establishment and use of third-party and front groups.^58-61^ They also reframed their corporate goals in terms of ‘harm reduction’ to renormalise,^61-65^ investing in alternative tobacco products, such as heated tobacco products and vapes.^61,66,67^ Such adaptivity is also apparent in the “greenwashing” undertaken by the fossil fuel industry^68^ and in the lower strength alcohol products marketed by the alcohol industry.^69^ This adaptivity is therefore important to take into consideration when developing interventions to UCI influence on public health policy. In this context, by “interventions,” we mean deliberate and structured actions, strategies, policies or organisational arrangements designed to change the system to reduce UCI’s ability to influence public health policy, thereby facilitating the advancement of policies that more closely align with public health objectives.

 A key component underlying UCI influence is corporate power, which is conceived of in various ways. One such way is that it stems from “material” (ie, financial) and “ideational” (ie, ideas) sources that manifest in different forms, namely instrumental (eg, use of lobbying and access to policy-makers), structural (eg, controlling the policy agenda and shaping institutional rules), and discursive (eg, framing policy in free market terms and emphasising personal responsibility).^7,70-73^ Drawing on theories of power, Gilmore et al argue that UCI practices influence the global structures in which public health policies are made, such as capitalism, globalisation, asymmetrical governance arrangements, international trade and investment practices, and regulatory frameworks – that ensure rules and norms favour industry.^1,7,70-72,74-82^ This suggests a reinforcing system — or feedback loop — that is both influenced by, and facilitates, UCI engagement in political practices. Such a system makes it challenging to advance effective public health policies, such as guidelines for consumption, or restrictions on the marketing, availability, and affordability of, and access to, unhealthy commodities. Instead of these policies, UCIs argue for deregulation, co-regulation, or neoregulation (ie, where states restructure supply chains according to public-private management^83-85^), despite the lack of evidence that these promote and protect public health.^1,86,87^

 To advance public health policies, changes are needed to curtail UCI power^72,75^ and prevent or mitigate their influence in the system.^4,35,88-90^ Recent literature has begun to explore this topic by, for example, suggesting or documenting various strategies or governance mechanisms to counter UCI influence on public health policy.^88,90,91^ These mechanisms aim to increase transparency; disclose industry influence and conflict of interest; identify, monitor, and educate policy-makers and the public about industry’s harmful practices; and manage or prohibit interactions with industry.^88^ Although these changes are needed, research lacks the consideration of industry adaptivity and the other complexities surrounding UCI influence on public health policy, including the underlying political, economic and social structures^77,84,92^ that enable UCI to engage in political practices.

 Systems mapping is a systems thinking approach involving a process of visually depicting the interactions, relationships, and feedback loops within a system, thus facilitating a comprehensive understanding of a phenomenon’s elements, dynamics and complexity.^41,45,51,93^ These maps can help to clarify the relationships between disparate parts of a system, and in this case, the pathways through which UCIs influence public health policy.^49^ In so doing, systems mapping could make explicit these interlinkages, rendering them more understandable in ways that might help to address UCI influence on public health policy.^44,52,94,95^ Using participatory systems mapping methods, this study fills a gap by making transparent the specific interrelationship between various parts of the system.

 In this study we focused on tobacco alcohol, and ultra-processed foods as they are the major preventable risk factors for non-communicable diseases (NCDs),^96,97^ which account for approximately 71% (41 million) of global deaths per year.^98^ These risk factors cause metabolic changes in the human body, such as increased blood pressure, obesity, hyperglycaemia and hyperlipidaemia, which increase the risk of developing NCDs.^99^

 Although previous studies have argued that UCI influence on public health policy is part of a complex system,^3,36,37^ as far as the authors are aware, this study represents a first attempt at explicitly applying participatory systems mapping methods to this phenomenon, thereby making a needed methodological contribution.

## Methods

###  Study Design

 We conducted participatory systems mapping workshops to map the complex pathways through which UCIs influence policy. These workshops are interactive events where stakeholders collectively create a visual representation, fostering a holistic and shared understanding of a complex system.^38,39,43,46,49,51,95,100-102^ In this case, the workshop brought together participants with knowledge of UCI influence to review a preliminary map, and identify, comment on and refine the linkages between UCIs, political practices, underlying structures, and other key actors that enable such influence.

###  Development of Preliminary Map

 To create a starting point for the participatory systems mapping workshops, a preliminary systems map was developed (See Supplementary file 1) by synthesising two recent publications: (*i*) *The Lancet* commissioned conceptual model of the CDoH,^1^ and (*ii*) an evidence-based taxonomy of political practices used by UCIs to influence public health policy.^2^ The latter paper, in turn, drew significantly from Legg et al who developed a model of corporate influence on science.^103^ These papers represent the most recent thinking and conceptualisation of the intersection between CDoH and UCI political practices. This involved identifying key elements (ie, the tangible or intangible components that constitute a system)^44^ and interconnections (ie, the “relationship [between the elements] that hold the elements together”).^44^ These were extracted into a table to compare the differences or similarities between elements, and then inputted to Kumu. Kumu is an online visualisation tool for creating system elements and drawing connections between them to produce an interactive diagram or map depicting complex relationships between various parts of a system or network. Kumu was used here because it allows one to visually depict the various interactions and dependencies surrounding UCI influence, and it has features that allow the creators to customise the map for the project’s purpose.

 As a starting point, a ‘target element’ was placed at the centre of the map to represent the extent to which industry successfully ‘influences policy.’ An ‘outcome element’ was then placed below to signify the outcome of UCI influence (ie, *deregulation and regulation that favours industry *and in the final map, *implementation of UCI preferred laws, regulations, processes and norms*). The target and outcome elements helped to distinguish them from other elements and remind the reader that this is the central purpose of the map – to explore the complexities surrounding UCI influence on public health policy. Each element in the systems map represents a variable (ie, a factor that can range between high and low values). Elements were clustered and synthesised if necessary and interconnections were linked to ensure that they reflected the narrative of each of the two studies. This process of clustering and integrating elements led to the identification of key distinct (yet interconnected and interdependent) themes that appeared to lead to successful UCI influences on public health policy.

###  Participatory Systems Mapping

 This study adapted in-person participatory systems mapping workshops^95^ by conducting a series of online “small group” workshops.^104,105^ Two pilot workshops were conducted to test the workshop design and refine the preliminary systems map.

 Two weeks prior to the workshops, a digital copy of the preliminary systems map was shared with participants, including a description of elements and interconnections, so that participants could become familiar with the map to maximise the time available for discussions. Participants were also sent a workshop brief that provided the purpose of the study, workshop agenda and questions, and some basic systems thinking terminology. Participants did not come back with queries.

 Workshop activities were adapted from the Causal Mapping with Seed Structure scripts.^106,107^ Participants were asked to consider the preliminary systems map and whether any elements or interconnections needed amending, removing, adding, or clarifying. The facilitator [AB] guided the participants around the various sections of the preliminary map using an online whiteboard. Participants’ views were captured by asking them to insert their comments using the sticky note function in the corresponding location on the map, after which comments were discussed. Each workshop focused on parts of the map where stakeholders had expertise. Stakeholder’s contributions were treated equally. Participants were also able to review, comment on and discuss other parts of the systems map if they felt they had insights to contribute. Workshops ranged between 60 minutes and 90 minutes in duration, were conducted on Microsoft Teams and were recorded and transcribed by the application. The transcriptions were then checked and amended, if necessary, by [AB, AvdA, or SD].

###  Participant Recruitment

 In an attempt to gain diversity of insights and perspectives, participants were purposefully sampled^108^ from a variety of backgrounds [academia (A), civil society (CS), former public officials (FPO), or global governance organisations (GGO)], covering different knowledge areas (tobacco, alcohol and ultra-processed food industries and/or CDoH), including all WHO geographical regions, and policy levels (eg, regional, national, and global).

 Stakeholders and their expertise were initially identified through a literature review and authors’ networks, and then through snowballing. Participants were selected if they had conducted research in the field of public health policy, UCI influence, or tobacco, alcohol or food policy, or issues underlying political, economic and social structures in which UCIs function; engaged in or represented organisations that engage in activities directly related to public health advocacy and awareness campaigns concerning tobacco, alcohol or food or other reform efforts concerning the CDoH; experience in policy-making or regulatory roles with a focus on health and industry influence issues; or involvement in global health governance or provided policy advice to national governments. Industry representatives were excluded because it was considered that they would have a conflict of interest.

 A total of 83 email invitations were sent to stakeholders. Fifteen declined to participate and 16 were unresponsive. Fifty-two stakeholders agreed to participate from different WHO regions (Africa = 17, Americas = 17, South-East Asia = 4, Europe = 27, Eastern Mediterranean = 1, and Western Pacific = 15) (See Supplementary file 2). Twenty-three small group workshops were conducted ranging between one and five stakeholders each between November 2021 and February 2022. Consent to participate was obtained from all stakeholders. They were also all given the option of being openly acknowledged for taking part in the workshops.

###  Analysis

 Workshop analysis involved reviewing workshop data (ie, workshop notes, whiteboard comments, and transcripts) in NVivo. Elements were extracted from the workshop data into a table to constantly compare their conceptual differences or similarities and how they interacted with each other. If a participant suggested that an element was important to UCI influence, it was included in the map. If there were to be a new element, it was inputted onto Kumu. Elements were then either amended, or new elements were added, clustered (if there were repetitions of the same or similar concepts), and then integrated if needed. Connections were drawn between elements to reflect participants’ views on the interrelationships between elements. Analysis did not assess the quantity or strength of evidence supporting each element or connection.

 Elements that are directly connected to the target element — we call “proximal elements” — represent the key issues that enabled UCIs to influence policy decisions. Elements were identified and connected to the proximal elements when stakeholders suggested what enabled these preceding elements. The entire analysis process was repeated until elements formed thematic clusters. Themes were named after proximal elements to represent the cluster of elements that were most closely interconnected. Illustrative quotes were used to embody the elements and resultant themes.

 The lead author emailed participants for clarification if needed. Once the analysis of workshop data was completed, a draft version of the full systems map, and a table describing the systems elements and interconnections were sent to all stakeholders for final sense checking. Responses to the final systems map were received from 14 participants, of whom 10 provided detailed feedback and four indicated they had nothing further to add. Feedback was then integrated into a final systems map.

## Results

###  System Overview

 The preliminary systems map (Supplementary file 1) consisted of 40 elements across six interconnected themes. Each theme symbolised a pathway through which UCIs influence public health policy. As the main purpose of this study is to present the final map, changes to the preliminary map are provided in Supplementary file 1.

**Figure 1:**
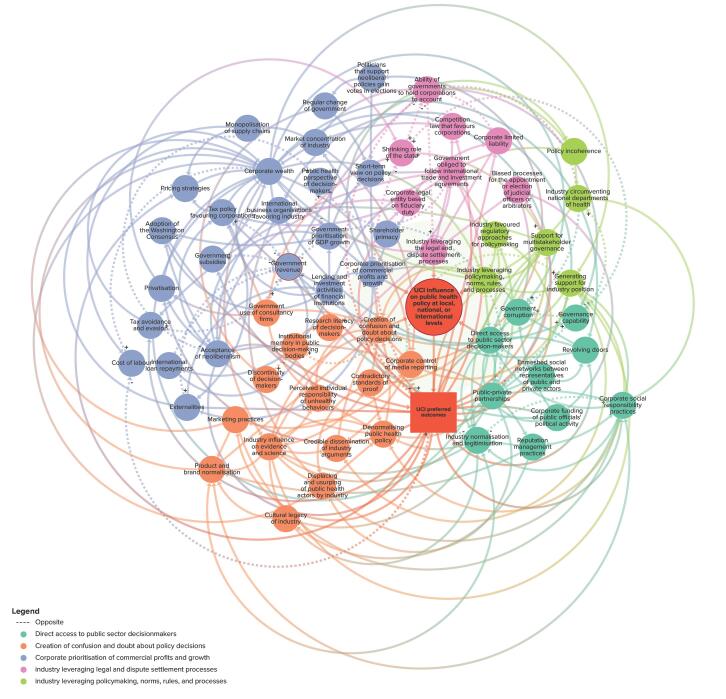
Full Systems Map. The solid lines represent the same direction of change between elements (eg, an increase in element A leads to an increase in element B. The dotted lines with a plus (+) or minus (-) sign represent an opposite direction of change between elements [eg, increasing (+) element A decreases (-) element B or vice versa]. There are also interconnections that are mutually directed ie, the arrows go in both directions, where an increase in one element leads to an increase in the other and vice versa. Abbreviation: UCI, unhealthy commodity industry.

**Figure 2:**
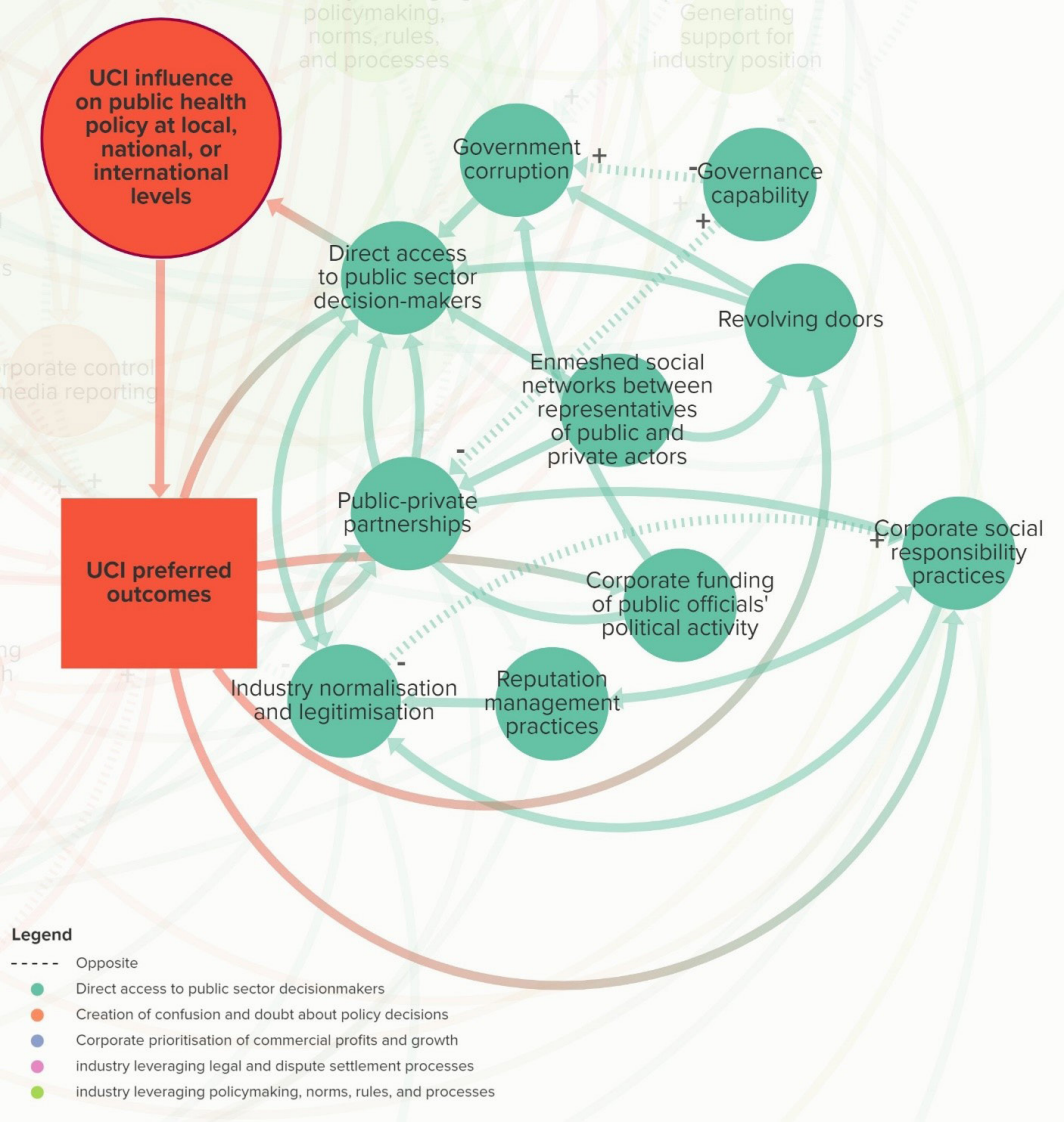
Direct Access to Public Sector Decision-Makers. Abbreviation: UCI, unhealthy commodity industry.

**Figure 3:**
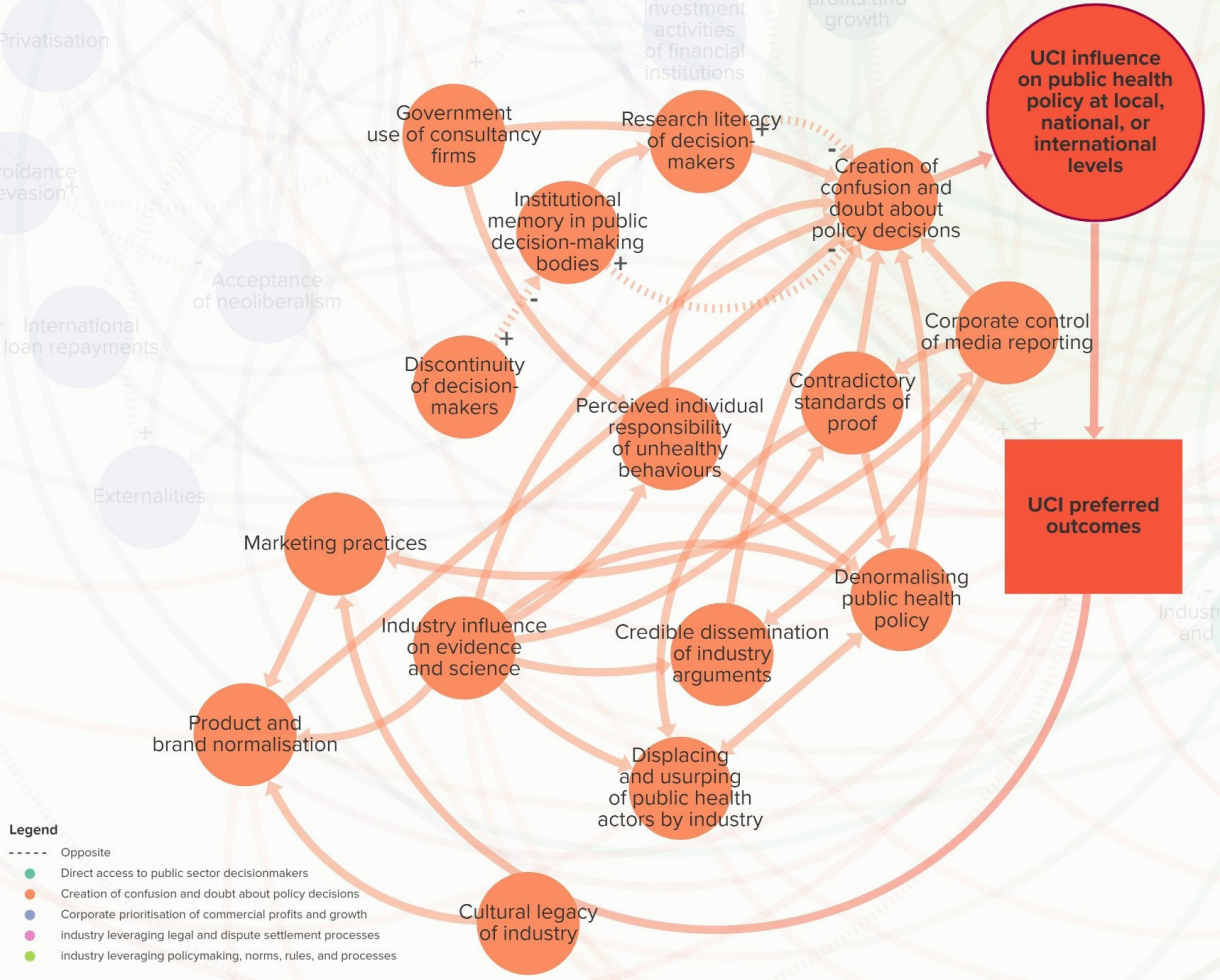
Creation of Confusion and Doubt About Policy Decisions. Abbreviation: UCI, unhealthy commodity industry.

**Figure 4:**
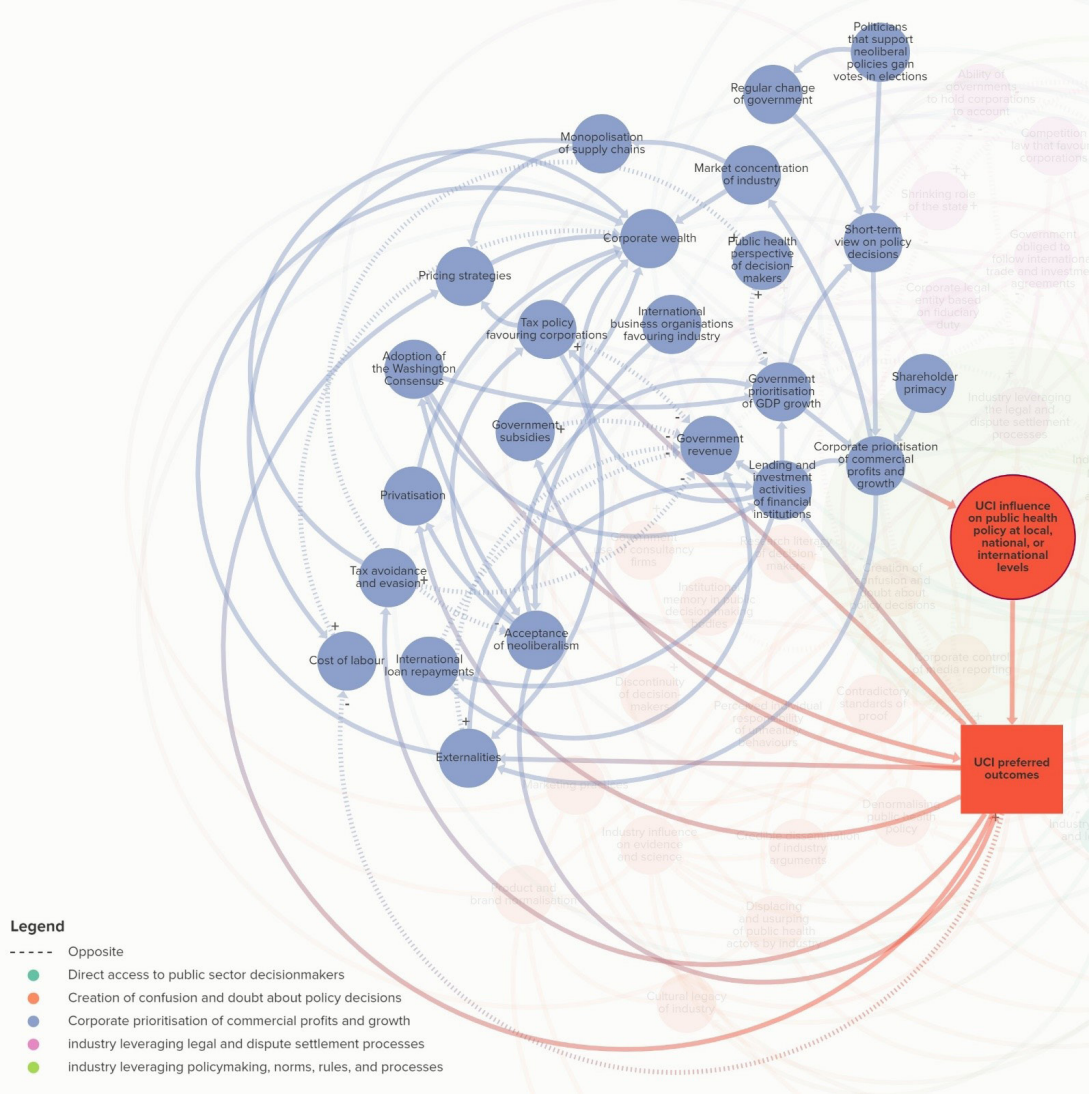
Corporate Prioritisation of Commercial Profits and Growth. Abbreviation: UCI, unhealthy commodity industry.

**Figure 5:**
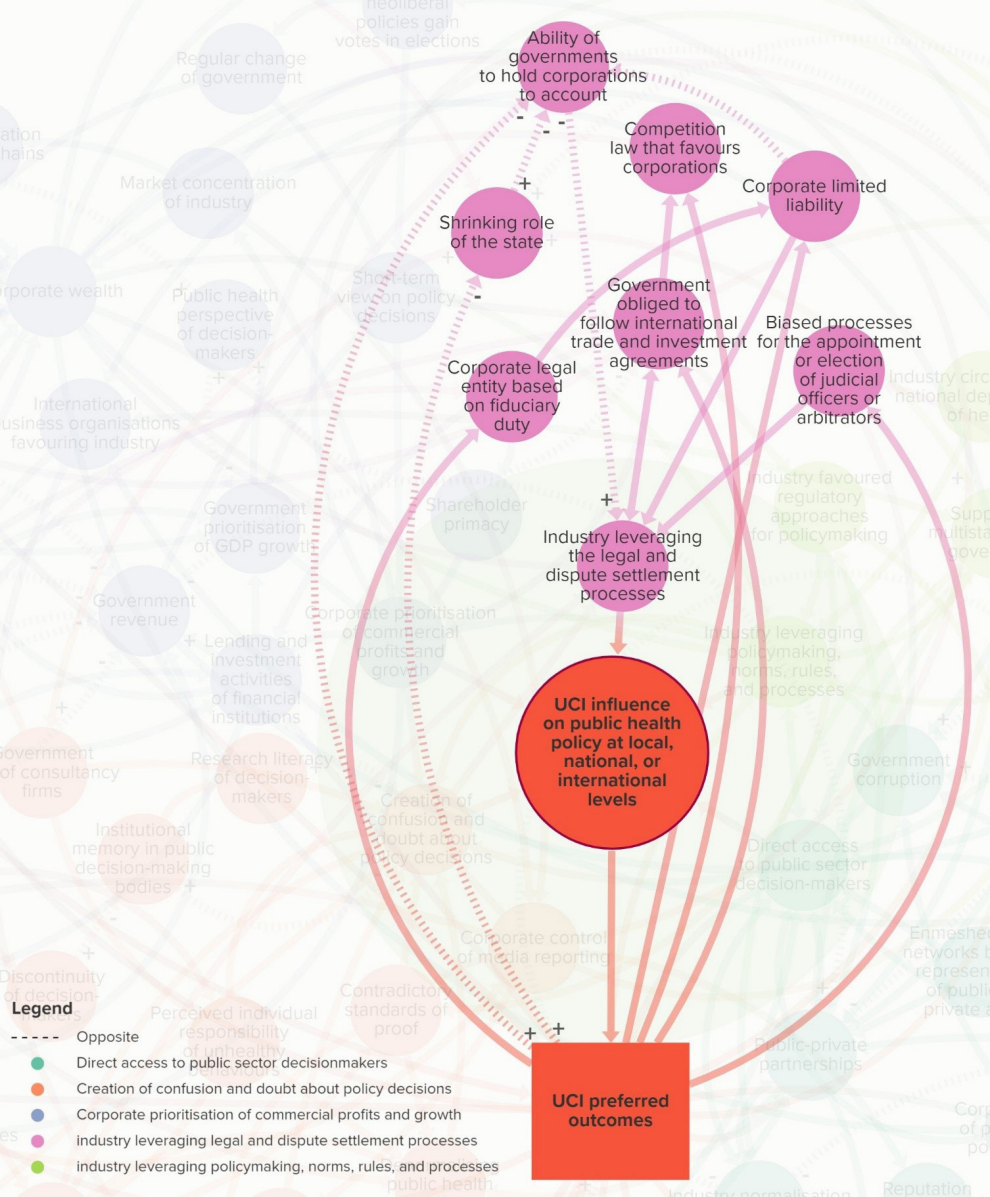
Industry Leveraging Legal and Dispute Settlement Processes. Abbreviation: UCI, unhealthy commodity industry.

**Figure 6:**
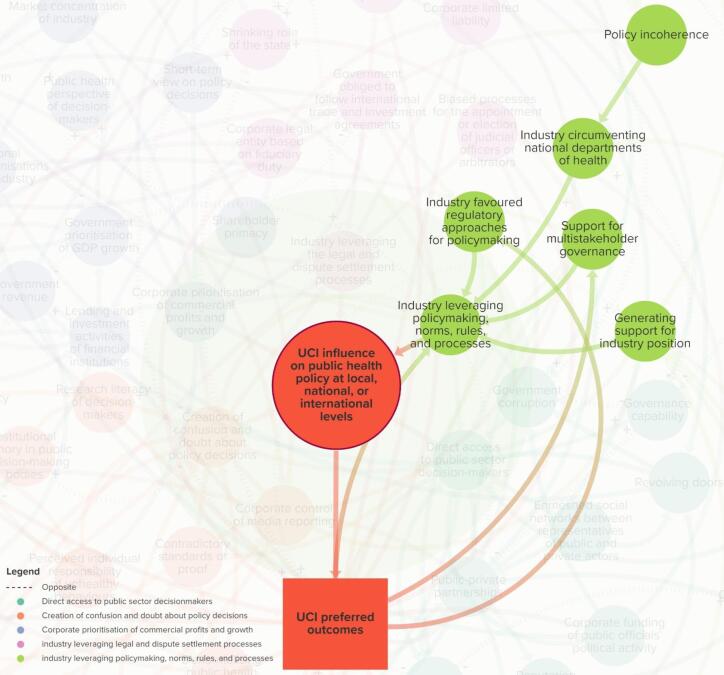
Industry Leveraging Policy-Making, Norms, Rules, and Processes. Abbreviation: UCI, unhealthy commodity industry.

 The final map consists of 64 elements across five themes (Figures 1–6 and online), namely: *direct access to public sector decision-makers *(Theme 1); *creation of confusion and doubt about policy decisions *(Theme 2); *corporate prioritisation of commercial profits and growth *(Theme 3); *industry leveraging legal and dispute settlement processes *(Theme 4); and *industry leveraging policy-making, norms, rules, and processes *(Theme 5).

 The “target element” (large red circle) represents the extent to which UCIs influence (ie, suppress, shape, weaken, block, or delay) policy at subnational, national, regional, or international levels. This leads to policies that favour UCIs — represented by the red rectangle (*UCI preferred outcomes*) — which in turn, connects to various pathways that are dispersed throughout the system. The proximal elements that connect directly to the large red circle — UCI influence on public health policy — are key pathways, and each element theoretically varies in value. A full list of element descriptions and their interconnections are provided in Supplementary file 3.

###  Theme 1. Direct Access to Public Sector Decision-Makers 

 The elements in Theme 1 affect the extent to which public sector decision-makers (policy-makers, civil servants, and public officials) can be directly accessed by industry actors (Figure 2). Stakeholders noted that industry achieves this through both formal (eg, being part of a policy committee) and informal (eg, through interpersonal relationships) means.

 Some of the key elements that affect direct access to decision-makers include *government corruption *(ie, extent to which corruption is endemic in a government), *revolving doors *(ie, extent to which individuals move between public office and industry jobs), and *enmeshed social networks between representatives of public and private actors* (ie, extent to which public sector decision-makers’ social networks are intertwined with senior corporate management^35^).

 Stakeholders noted that *government corruption* may feature more prominently within certain contexts. For example, *“[in] South Africa, we have a sort of different experience…corruption is endemic, but corruption of public officials is a big issue, so the officials who manage decision-making are very vulnerable to influence…*” (A13). *Corporate wealth* was also an important and interconnected element for this theme, “…*money is such an enabler for all of this, when you can buy big public affairs agencies and you can buy big comms campaigns and you’ve got access to the underground lobbying places…money buys access in multiple ways*” (CS16). Similarly, another stakeholder said, “*they have a ‘just-pick-up-the-phone’ kind of relationships that NGOs and civil society organisations wouldn’t even dream of having. But it’s because they’re normalised that this is an actor we have to consult. They’re at the table..*.” (FPO2). This suggests that close interpersonal relationships between decision-makers and industry representatives, together with industry as a normalised governance stakeholder, facilitated direct access to decision-makers.

 Within this theme, stakeholders also suggested that there was a reinforcing interaction between *public-private partnerships* (ie, extent to which public and private sector actors enter into partnerships) and *industry normalisation and legitimation* (ie, extent to which an industry is seen as socially acceptable and thus a legitimate policy actor). For example, a stakeholder said, “*…it’s almost like a sort of…vicious circle… the industry’s image is of a normal and legitimate policy actor. Therefore, it can be involved in public-private partnerships and governments mechanisms, which then reinforces its image*” (A23). Another stakeholder said, “*they’re actually sitting on advisory groups and panels around how to devise the policies and what’s acceptable to their members…we could never possibly even feasibly think about doing something without talking to industry and having them at the table” *(FPO2). This indicates that norms have shifted thus far in favour of UCIs that it does not need to expend political capital to secure access to policy-makers.

###  Theme 2. Creation of Confusion and Doubt About Policy Decisions 

 Elements in Theme 2 affect the extent to which decision-makers, and the public are confused about whether the proposed policy is needed and will lead to public value (Figure 3). Key elements that affect creation of confusion and doubt about policy decisions include *industry influence on evidence and science*^2,103^ (ie, extent to which industry funds, produces, controls, and manages information and research); *displacing and usurping of public health actors by industry*^2,103^ (ie, extent to which industry attempts to marginalise and take over the role of public health actors); *denormalising public health policy*^2^ (ie, extent to which industry creates the perception that public health policy interventions are unnecessary or socially unacceptable); and *perceived individual responsibility of unhealthy behaviours* (ie, extent to which individuals are perceived as being responsible for their own patterns of consumption behaviours).

 Stakeholders pointed out the complex dynamic between elements, such as *marketing practices *(ie, extent to which marketing is unrestricted and poorly monitored and enforced), *industry normalisation and legitimisation, *and *product and brand normalisation* (ie, extent to which a product is seen as socially acceptable or desirable). For example, one stakeholder said, *“[industries] promote brands rather than products. But using products in a way that normalises the product so… they can use brands instead of products … because when you advertise to children, you’re not supposed to advertise unhealthy things, so you don’t advertise the actual product, you just advertise the unhealthy brand. And sometimes the brands have healthy and unhealthy foods under them, so it creates confusion*….” (A15). This suggests that corporate branding is a means to normalise an industry, making it challenging to refute their claims and arguments and enabling access to decision-makers.

 Stakeholders also highlighted the complex relationship between *industry influence on evidence and science; corporate control of media reporting *(ie, extent to which corporations control what is reported in the media through, for example, media ownership and marketing or sponsorships); *credible dissemination of industry arguments *(ie, extent to which industry creates a perception of producing credible information, disseminated through networks of actors); and *generating support for industry position* (ie, extent to which corporate actors fabricate or galvanise support with stakeholders) (Theme 5). For example, a stakeholder noted, “*media reporting will kind of pick up on these kinds of oversimplistic arguments and not really interrogate them further…maybe that’s because of who owns the media outlets” *(CS15). Another stakeholder noted that “*[industries]…are big spenders and big sources of income for media in terms of advertising in all sorts of media…that might also discourage the journalists within those media to just put the critical light on these industries” *(CS4). Moreover, another stakeholder said, “*[when industry] get a bit of bad publicity, they change their name… But they’re back again…but on front groups [sic] and other funded organisations, which include [their funded] research” *(A26). Stakeholders suggest that underlying the confusion and doubt about policy decisions is the cumulative impact of industry research disseminated through biased media, thereby entrenching industry support.

 In response to *contradictory standards of proof *(ie, extent to which evidentiary standards of proof are applied inconsistently to industry and public health actors and evidence), a stakeholder remarked, “*we’re held to these ridiculously high standards of evidence that just doesn’t affect the other side *[industry]*”* (CS16). The same stakeholder suggested that *discontinuity of decision-makers *(ie, extent to which public officials remain in their positions as decision-makers over time) was an important aspect that was linked to *institutional memory in public decision-making bodies *(ie, extent to which a decision-maker represents a public institution over time), “*… voluntary reformulation programme on sugar [industry] hasn’t worked. Yet the government is still going down this path because it’s a different set of politicians who think, ‘well, we can make it work because we’re not like those ones who did it and got it wrong’”* (SC16). This indicates that confusion and doubt can be worsened by a lack of knowledge on effective public health measures.

###  Theme 3. Corporate Prioritisation of Commercial Profits and Growth

 Elements in Theme 3 affect the extent to which corporations prioritise their own profits and growth above other economic costs associated with consuming unhealthy commodities (or other societal values, such as health, well-being, human rights, and the natural environment). Some of the key elements that affect this element are *shareholder primacy* (ie, extent to which corporations maximise the value of their company for shareholders); *government prioritisation of GDP *[gross domestic product]* growth *(ie, extent to which government prioritises the notion of GDP growth as a measure of economic growth); *lending and investment activities of financial institutions *(ie, extent to which public and private financial institutions provide capital for UCIs);and *short-term view on policy decisions *(ie, extent to which politicians adopt a short-term view on policy decisions).

 Stakeholders mentioned the *externalities* of costs and the need to structure the economy by using taxes* “…to better reflect the externalities…”* (CS11), is linked to how UCIs use arguments to “*emphasise the benefits that they bring [to economic growth] but hide completely the costs…” *(A8). Although industries make these economic arguments, stakeholders noted that the converse was the case, *“NCDs in general and alcohol harm… undermine economic growth. So, the harm, the costs of the harm…are…bigger than what the industry brings in through tax revenue*” (CS6). Similarly, another stakeholder said, *“*…[the] *choice between public health and the good economy is a completely [sic] false dichotomy, because clearly a healthier workforce would be a more productive workforce”* (CS11). Additionally, to make the link that *shareholder primacy *is an important part of this theme, a stakeholder said, “…*it’s not just the ideology of economic growth...the wealthiest – like the shareholders – [is] really where the wealth is accumulated” *(CS6).

 Stakeholders suggested a relationship between *government use of consultancy firms* and *privatisation*, which is related to the *shrinking role of the state,* and *governance capability*. For example, a stakeholder said, “*we’re now outsourcing government policymaking to a consultancy firm to write a policy for government. And of course, when you look at what they write, they often recommend privatisation, which, they have such a strong interest in. So, there is this kind of perfect loop for the corporation between recommending privatisation and then public sectors get deskilled*” (A9). Moreover, in discussing the relationship between *corporate prioritisation of commercial profits and growth* and the *creation of confusion and doubt about policy decisions,* a stakeholder noted that “*the failure to achieve growth is an important story…” *(A4). Thisfinding alludes to industry arguments or narratives that regulations on UCIs inhibit economic development, which creates doubt about advancing public health policies.

 It is important to note that some stakeholders challenged the element *acceptance of neoliberalism *(ie, extent to which governing bodies subscribe to the political ideology of market fundamentalism^1^). For example, a stakeholder said that they were, “*uncomfortable with use of terms like ‘neoliberalism’*” because it “*implies a partisan political dimension*” (A26). Similarly, another stakeholder suggested that the term ‘neoliberalism’ was divisive as it alienated policy actors who required it as a fundamental prerequisite for international cooperation — for example, in trade agreement negotiations, “*so we can’t point fingers at the whole concept of free trade agreements, but we could say that fine, free trade agreements could provide a lot of benefit, but definitely not for products that have zero benefit like tobacco…*” (CS17). In contrast, another stakeholder said, “*neoliberalism is not even perceived as an ideology anymore – and I feel that is the most powerful form of ideological persuasion – is you simply take it off the table as a subject*” (A4). This indicates the view that the term ‘neoliberalism’ is also important to retain in the system maps so that it continues to be a topic for discussion and debate.

###  Theme 4. Industry Leveraging Legal and Dispute Settlement Processes

 Elements in Theme 4 affect the extent to which industry leverages legal and dispute settlement processes, for example, by bringing or threatening to bring litigation against governments to prevent, undermine, or reverse public health policy. Some of the key elements that affect these processes are *biased processes for the appointment or election of judicial officers or arbitrators*; *government obliged to follow international trade and investment agreements; ability of governments to hold corporations to account* (ie, extent to which governments have the ability to investigate, prosecute and sanction problematic corporate behaviours in domestic and extra-territorial jurisdictions),and *corporate limited liability* (ie, extent to which the owners or management of commercial entities are liable for corporate debt, damages, or wrongdoing).

 For the preliminary map, the elements pertaining to international trade and investment agreements were initially itself a proximal element. However, stakeholders suggested that it should be integrated within Theme 4 because how industry influences international trade and investment agreements are the same pathways that lead to the target element (large red circle). For example, one stakeholder said, “*when I was thinking about how the industry lobbies through… direct access – so they have direct access to policy-makers nationally, but also in the regional…and international trade agreements...*” (A2). Stakeholders also highlighted the importance of unpacking what lay within international trade and investment rules, norms and processes. Stakeholders suggested connecting *competition law that favours corporations* (ie, extent to which competition law favours corporations, including mergers and acquisitions and intellectual property), with *government obliged to follow international trade and investment agreements,* and *monopoly concentration*. For example, a stakeholder noted, “*competition policy…gets used in trade and investment agreements…”* (A1) and that “*when you’re talking about food, alcohol, and tobacco, we see mergers and acquisitions happening across those sectors*” (A1). Similarly, a stakeholder said, “*monopoly concentration is a really important dynamic of the last few decades that has significantly increased the power of industry..*.” (CS6), which shows how UCI’s legal structures help to entrench their trade and economic power.

 Another important concept stakeholders noted was the effect of industry bringing, or threatening to bring, litigation, which causes regulatory chill. It is a powerful deterrent for governments developing public health policy, as one stakeholder said, “*in terms of governments not wanting to try something because they fear litigations or they fear repercussions in related to their trade agreements – so even without governments pushing or the industry asking their governments to raise concerns with other governments, they still might hold back on regulations due to the fear of this happening*” (CS4). Similarly, another stakeholder said, “*we don’t know what the chilling effect is on public health legislation simply because people are scared of being sued*…” (A9). Lastly a stakeholder noted, “*leveraging the legal system… all comes down to budgets, we’re aware that a big multinational food company is bringing a legal challenge*”(CS16). This highlights, as noted above, that *corporate wealth* is a crucial element that leads to various other elements, including using UCI resources to take legal action.

###  Theme 5. Industry Leveraging Policy-Making, Norms, Rules, and Processes

 Elements in Theme 5 affect the extent to which industry leverages national and international policy-making norms, rules and processes that favour their participation in policy-making. Some of the key elements that affect these are* industry favoured regulatory approaches for policy-making *(ie, extent to which governments establish regulatory approaches that mandate industry favoured policy-making procedures, such as business impact assessments, stakeholder consultations, and risk assessments^109^); *industry circumventing national departments of health; support for multistakeholder governance *(ie, extent to which national and global governance institutions support the norm for including non-public sector stakeholders in decision-making processes, including corporations); and *generating support for industry position.*

 Stakeholders suggested that UCIs leverage the norm on multistakeholder governance by arguing for the involvement of UCIs in policy-making processes, which is now incumbent upon decision-makers to fulfil. In this theme, participants suggested that UCIs therefore do not necessarily need to use “political practices” to influence public health policy, but the predominant norm for decision-makers is to always involve industry in policy decisions, despite potential conflicts of interest. For example, a stakeholder said, “*…when I consult with governments, they say that because of the… [Sustainable Development Goal 17, SDG 17] we have to involve industry and we have to follow the SDGs*” (GGO1). The same stakeholder said, “*…industry does not need to lobby at all…[public-private partnerships] are sold without any evidence. It’s just the prevailing idea…”* (GGO1). Stakeholders also noted that industry involvement in policy processes is already normalised “…*they are a natural, normal part of the policy-making and policy implementation process*” (A23) and “*have this privilege sort of access over other kinds of ‘natural citizens’ into these decision-making processes*” (A23). Stakeholders noted that depending on the policy-making spaces, industry actors may go unchallenged due to the lack of CS actors representing public health interests; for example, in international trade negotiations, “*public health [actors are]…not in the WTO [World Trade Organisation]”* (CS6). Importantly, stakeholders suggested that industry was instrumental in creating this norm of industry involvement in policy discussions, “*the private sector was involved in the development of the SDGs*” (GGO1) and industries argue, “*… ‘well, but we live in a democracy. We are a social actor just like anybody else. Why can’t we participate in the decision-making process?*’” (CS9). This suggests how UCIs contribute to developing the frameworks in which policy is made, thus creating feedback loops that perpetuate the ability of UCIs to influence policy.

 Stakeholders suggested that there is a disparity between government departments specifically those involved in the economics or finance. For example, a stakeholder said, “*government finance, the treasury really does have a veto over [other departments]… They really do control everything across government departments*” (FPO4). This is represented by the linked elements *industry circumventing national departments of health,* and *policy incoherence* (ie, extent to which there are inconsistent policy goals between government departments).

 Stakeholders acknowledged that at the international level, industry is easily represented in policy-making, such as when negotiating trade and investment agreements, whereas this is not the case for public health representatives, “*the balance between industry and public health influence on trade agreements [is] very hard for public health people to get on the agenda, whereas industry has an open door in those trade agreements*…” (A9). Similarly, stakeholders said, “*trade is really like a competing policy arena compared to global health*” (CS6), and “*industry and trade policies tend to trump out the public health concerns*” (CS4). This indicates that trade issues crowd out public health concerns in policy-making spaces.

## Discussion

 This study aimed to build a systems map depicting the complex pathways through which UCIs influence public health policy. As such influence exhibits characteristics of a complex system, a systems map could help to visualise this complexity, thereby helping to identify ways to change the system. Although previous studies have suggested that UCI influence are complex systems problems,^1,3,36,37^ to the best of the authors’ knowledge, this study represents the first attempt to explicitly apply participatory systems mapping methods to understand this phenomenon, thus making a methodological contribution. Importantly, this map is neither intended to be comprehensive nor exhaustive, but seeks to provide a starting point for applying systems thinking to explore the complexities surrounding this problem.

 We identified five distinct, yet interdependent, themes through which UCIs influence public health policy, which collectively comprise a complex web of interconnected and diverse underlying structures dispersed throughout the system. UCI influence therefore does not have a single point of origin, but is inextricably linked to different parts of the system. This suggests that reducing UCI influence may require disrupting more than one pathway, as is suggested by those within systems science.^38,44,45,52,95^

 One interesting implication from this work is that maps such as these can be used to demonstrate the many interconnections between parts of the system and thus the range of pathways industry could adapt to change (as is known to have occurred after the implementation of Article 5.3, mentioned above^58-67^). As a hypothetical example, although a change may aim to reduce UCIs’ direct access to public sector decision-makers, UCIs may generate support for their position using front groups to access policy-makers on their behalf. Broader lobbying regulations and transparency policies should therefore be implemented to ascertain whether front groups represent UCIs to manage their access. This systems map helps to make this complexity apparent; it could help to better predict how the system responds to changes,^41,51^ demonstrate why some changes may be unpredictable^110-112^ and lead to unintended outcomes.^41,44,113^

 Importantly, while this map offers valuable insights into the complexity surrounding UCI influence, some parts of this map may be less relevant in specific settings, such as authoritarian regimes^114^ versus liberal democracies,^115^ or free markets^116,117^ versus mixed or controlled economies.^117,118^ For example, given the global prioritisation of GDP growth as a key driver of development,^119^ Theme 3 (…*prioritisation of commercial profits and growth*) may be more widely applicable. Conversely, given that varying degrees of authoritarianism and freedom of expression may control the flow of information,^120^ Theme 2 (*creation of confusion and doubt…*) may be less applicable in some contexts.

 A key question is whether UCI influence is a feature, or a bug, of these underlying structures. If it is a feature, to address UCI influence on public health policy, global society needs to be prepared to change these structures. If society is not prepared to do this, then we need to come to terms with the fact that UCIs will pose a constant barrier to developing effective public health policy, and we need to be realistic about how effective a limited set of interventions can actually be. If, however, it is a bug, then the question is what are the necessary and sufficient conditions to prevent and mitigate UCI influence on public health policy without changing these underlying structures?

 This map also depicts how corporate power manifests in different parts of the system. Consistent with the public health literature, this map shows the pathways in which the sources of power (material and ideational) are manifested into instrumental, structural and discursive forms of power, and the overall feedback in which corporate power is perpetuated.^7,70-73^ UCI influence is clearly a cumulative outcome of these power asymmetries that are upheld, in large part, by the current global political-economic system, which is a key driving force behind the prioritisation of economic/trade policies over public health goals.^84,92,121-127^ Researchers have suggested leveraging the economic/trade sectors and supply chain actors^84,121,125,128,129^ to incentivise a system toward prioritising public health outcomes.^130-132^ However, there may be a fundamental conflict between aspects of economic/trade goals and public health goals,^92^ which system changes may need to focus on resolving, such as ways of measuring economic growth,^92,133,134^ the legal status of corporations,^8^ and UCI fiduciary duties to maximise profits for shareholders.^135^

 Consistent with the literature, this systems map shows the interdependence between different parts of the system. For example, governments are obligated to abide by competition law (including intellectual property protections) through international trade and investment agreements […*legal and dispute settlement processes *(Theme 4)], leading to the market concentration of UCIs^136-138^ and dominance over their supply chains^137,139-141^ […*prioritisation of commercial profits and growth *(Theme 3)]. Similarly, the literature suggests that UCIs use market-based practices (ie, business practices) to gain a competitive advantage in the market,^74,142,143^ increasing market concentration.^74,75^ However, the literature does not always make apparent — which this map helps to do — the cumulative impacts of factors, such as poor labour laws,^144,145^ pricing strategies,^146,147^ tax cuts,^148^ tax avoidance and evasion,^149^ government subsidies,^150,151^ intellectual property laws,^1^ and externalities.^152^ These together enable corporations to amass a vast amount of wealth, which they, in turn, use to fund their political practices,^8,74,75^ thus perpetuating a feedback loop between UCI political practices and underlying structures.

 Other independencies between systems mapping themes are consistent with the literature. For example, arguments by UCIs in policy consultations at international forums, such as the WHO and WTO, and in national forums […*policy-making norms, rules, and processes *(Theme 5)], where they use ideological precepts of neoliberalism […*prioritisation of commercial profits and growth *(Theme 3)] to downplay UCI responsibility in individuals’ consumption of unhealthy products^77,153-155^ [*creation of confusion and doubt… *(Theme 2)]. Moreover, the literature illustrates how UCIs use both the threat of litigation and the act of litigating in both domestic and international courts (ie, Investor-State Dispute Settlements) as a long-term strategy to delay the implementation of policy and as a means to achieve regulatory chill.^137,156-159^ In such spaces they may create confusion and doubt by, for example, attacking evidence that support public health measures^156^ or arguing that regulations will have a negative impact on smaller businesses, or disproportionately impact vulnerable groups.^157^

 Lastly, UCI influence on public health policy is inextricably linked to how they attempt to influence science, which is a powerful source of industry discursive strategies (ie, industry arguments) for creating confusion and doubt about the harms of their products, legitimising their role as key contributors to science, and advocating for their preferred policy solutions.^103^ Similarly, UCIs use their involvement in the production of evidence and science to make equivalent arguments in policy processes at national and international levels,^29,35,103,154,155,160^ which are compounded by industry favoured regulatory approaches, such as the use of impact assessments, stakeholder consultations, and risk assessments — as seen in “Better Regulations” approaches in the UK and EU^109,161^ […*policy-making norms, rules, and processes *(Theme 3)]. These regulatory approaches are easily dominated by UCIs, which frame issues in terms of economic or financial costs as opposed to social or health costs.^109,161^

###  Strengths and Limitations

 This study gathered insights from diverse stakeholders from varied geographic backgrounds and expertise to balance different perspectives. Despite this diversity, stakeholders generally agreed with the broader pattern of UCI influence on public health policy, and the cumulative impact of multiple interacting elements that enable such influence. This study achieves a unique level of inclusivity and representation to ensure a comprehensive understanding of the system, enriching the depth and breadth, and enhancing the credibility, of the results. Additionally, the participatory nature of the workshops foster a sense of ownership and collaboration among stakeholders, making them active contributors to the research process.^162^

 There were also several limitations to this study. Firstly, the systems map is subjective, based on purposively sampled stakeholders, the theoretical lens and knowledge of the facilitator,^95,163^ and provides only a static visualisation. Although this study achieves a diverse geographical spread, some regions were overrepresented (eg, Europe) whereas others were underrepresented (eg, the East Mediterranean), which may have affected the overall generalisability of the map to different countries and jurisdictions. It is likely that the map will change depending on the period it is captured, the sample of participants, and as different perspectives and knowledge are incorporated. Importantly, this map also does not capture the quantity of evidence supporting each element or the strength of the links between elements. Lastly, participants’ input was welcomed and given equal weight if they felt that they could provide helpful input. This may have adversely affected the map as some participants may have limited knowledge on some parts of the system in which they contributed. However, in practice, participants generally provided input into the areas for which they were selected. Secondly, there was also a broad range of views regarding the language in which to frame elements, the need to balance element granularity (more detail) versus integration (less detail) and its generalisability across context versus being context specific. Indeed, it is important to note that the map depicts broad patterns across different industries and contexts and, as such, a degree of interpretation is needed when applying the map to specific cases.

 Thirdly, although this study successfully conducted small group participatory systems mapping workshops for the online environment, it would have benefited from in-person workshops. Stakeholders between workshops were unable to engage with each other directly, which was mitigated by the facilitator relaying ideas across groups. Moreover, stakeholders did not always make themselves clear in workshop discussions. Analysis at times needed to rely on interpreting stakeholder meanings, making it challenging to capture participants’ views and translating them into a systems map. This was mitigated by emailing participants for clarification and seeking feedback from participants on the systems map — although the map would have benefited from further and repeated participant input.

 Finally, when stakeholders prepared for workshops by reviewing the preparatory materials, workshop discussions were richer and more constructive. As some stakeholders did not always prepare for workshops, some discussions became stagnant, or more time was needed to explain systems mapping concepts.

## Conclusion

 This systems map helps to communicate the complexity of UCI influence, namely how pathways to influence are interconnected with each other and their underlying political, economic and social structures. This complexity poses challenges for formulating a singular intervention or limited set of interventions capable of effectively countering such UCI influence. To further help identify areas for systems change, future research could refine or condense this map. Elements or connections could also be weighted, or one may develop a systems-dynamic-model to vary inputs to show which strategies may have more impact on limiting UCI influence. Additionally, to help understand the differences in UCI influence in particular contexts or jurisdictions, further research could adapt or directly apply this map as a conceptual framework in case studies investigating UCI influence, or for testing the relationship between factors that enable influence. Finally, to understand the differences and similarities between systems of influence, researchers could apply systems mapping methods to other industries that seek to influence public health policy in ways that negatively impact public health outcomes, such as the firearms, automobile, pharmaceutical, gambling, or extractive industries, and compare findings.

## Acknowledgements

 This article is dedicated to the memory of Mateusz Zatoński. Mateusz was a wonderful colleague, and his sad death leaves a gap in tobacco control research which will be hard to fill. He was committed to helping countries worldwide strengthen their tobacco control measures and improving public health for all. He will be much missed.

 The authors would like to thank all the participants for giving their time and sharing their invaluable expertise. Fully acknowledged participants are placed in alphabetical order: *Alice Fabbri*, Tobacco Control Research Group, University of Bath; *Andrew Rowell*, Tobacco Control Research Group; *Angela Carriedo*, World Public Health Nutrition Association; *Belinda Townsend*, Australian National University; *Ben Hawkins*, MRC Epidemiology Unit, University of Cambridge; *Britta Katharina Matthes*, Tobacco Control Research Group, University of Bath; *Caroline Cerny*, Obesity Health Alliance; *Charles DH Parry*, Alcohol, Tobacco & Other Drug Research Unit, South African Medical Research Council; *Crispin Acton*, former civil servant, United Kingdom; *Daniel Dorado*, Corporate Accountability; *Debby Sy*, Global Centre for Good Governance in Tobacco Control, a STOP Partner; *Dhamaravelli ( Vimla ) Moodley*, former civil servant South Africa; *Fran Baum*, Stretton Health Equity, University of Adelaide; *Hazel Cheeseman,* Deputy Chief Executive, ASH; *Jaime Arcila*, Corporate Accountability; *Jane Martin,* Obesity Policy Coalition; *Jennifer Lacy-Nichols*, University of Melbourne; *Kate Oldridge Turner*, World Cancer Research Fund International; *Kathrin Lauber*, University of Edinburgh; *Kathryn Reilly*, former member of Irish Parliament, *Leigh Haynes*, Framework Convention on Global Health Alliance; *Leslie London*, School of Public Health and Family Medicine, University of Cape Town; *Lilia Olefir*, NGO Advocacy Centre “LIFE”; *Lori Lake*, The Children’s Institute; *Lucy Westerman,* VicHealth; *Maik Dünnbier*, Movendi International; *Marian Lorena Ibarra*, Food Policy Program, Global Health Advocacy Incubator; *Mark Tomlinson*, Institute for Life Course Health Research, Department of Global Health, Stellenbosch University; *Martin McKee*, Professor of European Public Health, LSHTM; *Mateusz Zatoński*, Tobacco Control Research Group, University of Bath; *Mike Daube*, Curtin University, Western Australia; *Mindaugas Štelemėkas*, Head of Health Research Institute, Faculty of Public Health, Lithuanian University of Health Science; *Nason Maani*, Global Health Policy Unit, University of Edinburgh; *Nicholas Freudenberg*, CUNY School of Public Health; *Nina Renshaw*, NCD Alliance; *Øystein Bakke*, Global Alcohol Policy Alliance; *Patricia Lambert*, International Legal Consortium at the Campaign for Tobacco-Free Kids; *Paula Johns*, ACT Health Promotion, Brazil; *Pepita Barlow*, Department of Health Policy, LSE; *Peter Rice*, European Alcohol Policy Alliance; *Petronell Kruger*, The SAMRC/Wits Centre for Health Economics and Decision Science (PRICELESS SA); *Raquel Burgess*, Department of Social & Behavioural Sciences, Yale School of Public Health; *Rima Nakkash*, George Mason University; *Sarah Dance*, Tobacco Control Research Group, University of Bath; *Sebastian Rositano*, Commercial and Economic Determinants of Health Unit, World Health Organization; *Sharon Friel*, Australian National University; *Sulakshana Nandi*, Public Health Resource Network Chhattisgarh India; *Susan Goldstein*, SAMRC Centre for Health Economics and Decision Science PRICELESS, School of Public Health University of the Witwatersrand; *Tara Van Ho*, University of Essex; *William F Lamb*, Mercator Research Institute on Global Commons and Climate Change (MCC). The following participant prefer to be acknowledged by only their affiliation: Oregon State University. One participant preferred not to be acknowledged.

 Authors would also like to acknowledge Melissa Mialon and Mark Petticrew for early discussions prior to the development of the study, and Bryan Clift, Selda Ulucanlar, and Harry Rutter for their input on analytical methods.

## Ethical issues

 Ethical approval (EP20/21002) was granted by Research Ethics Approval Committee for Health (REACH), University of Bath.

## Competing interests

 The authors declare that they have no competing interests, financial or otherwise, related to the current work. To the best of our ability, this also includes the late Mateusz Zatoński.

## Funding

 AB received a PhD studentship from University of Bath. AG is supported by the UK Prevention Research Partnership (MR/S037519/1), which is funded by the British Heart Foundation, Cancer Research UK, Chief Scientist Office of the Scottish Government Health and Social Care Directorates, Engineering and Physical Sciences Research Council, Economic and Social Research Council, Health and Social Care Research and Development Division (Welsh Government), Medical Research Council, National Institute for Health Research, Natural Environment Research Council, Public Health Agency (Northern Ireland), The Health Foundation and Wellcome. KB is supported by the UK Prevention Research Partnership (MR/S037586/1). AVDA is funded through PhD funding from the University of Bath, in affiliation with the SPECTRUM consortium (MR/S037519/1). SPECTRUM is funded by the UK Prevention Research Partnership (UKPRP). SD and MZ were funded through Bloomberg Philanthropies’ Stopping Tobacco Organizations and Products funding (http://www.bloomberg.org/). None of the funders had any role in the study design, data collection and analysis, decision to publish or preparation of the manuscript.

## Additional Information

 Our dear co-author Mateusz Zatoński, PhD, sadly died on January 17, 2022.

## Supplementary files

Supplementary file 1. Preliminary Systems Map.

Supplementary file 2. Participant Knowledge Areas and Workshop Groups.

Supplementary file 3. Element Descriptions and Interconnections.
