# Supplementary material for: Building a Systems Map: Applying Systems Thinking to Unhealthy Commodity Industry Influence on Public Health Policy
Source: Int J Health Policy Manag. 2024 Apr 7;13:7872. doi: 10.34172/ijhpm.2024.7872 (PMC11607592; doi:10.34172/ijhpm.2024.7872)
Supplement: Supplementary file 1 — Preliminary Systems Map. [file ijhpm-13-7872-s001.pdf]

Article title: Building a Systems Map: Applying Systems Thinking to Unhealthy Commodity Industry Influence on Public Health Policy

Journal name: International Journal of Health Policy and Management (IJHPM)

Authors' information: Adam Bertscher[1]*, James Nobles[2], Anna B. Gilmore[3], Krista Bondy[4], Amber van den Akker[3], Sarah Dance[5], Michael Bloomfield[1], Mateusz Zatoński[3]

[1]Department of Social and Policy Sciences, Faculty of Humanities & Social Sciences, University of Bath, Bath, UK.

[2]Centre of Active Lifestyles, Leeds Beckett University, Leeds, UK.

[3]Department for Health, Faculty of Humanities & Social Sciences, University of Bath, Bath, UK.

[4]School of Management, Marketing, Business & Society, University of Bath, Bath, UK.

[5]Department of Psychology, Faculty of Humanities & Social Sciences, University of Bath, Bath, UK.

Additional Information: Our dear co-author Mateusz Zatoński, PhD, sadly died on January 17, 2022.

***Correspondence to:** Adam Bertscher; Email: ab3298@bath.ac.uk

Citation: Bertscher A, Nobles J, Gilmore AB, et al. Building a systems map: applying systems thinking to unhealthy commodity industry influence on public health policy. Int J Health Policy Manag. 2024;13:7872. doi:10.34172/ijhpm.2024.7872

Supplementary file 1. Preliminary Systems Map

Preliminary systems map

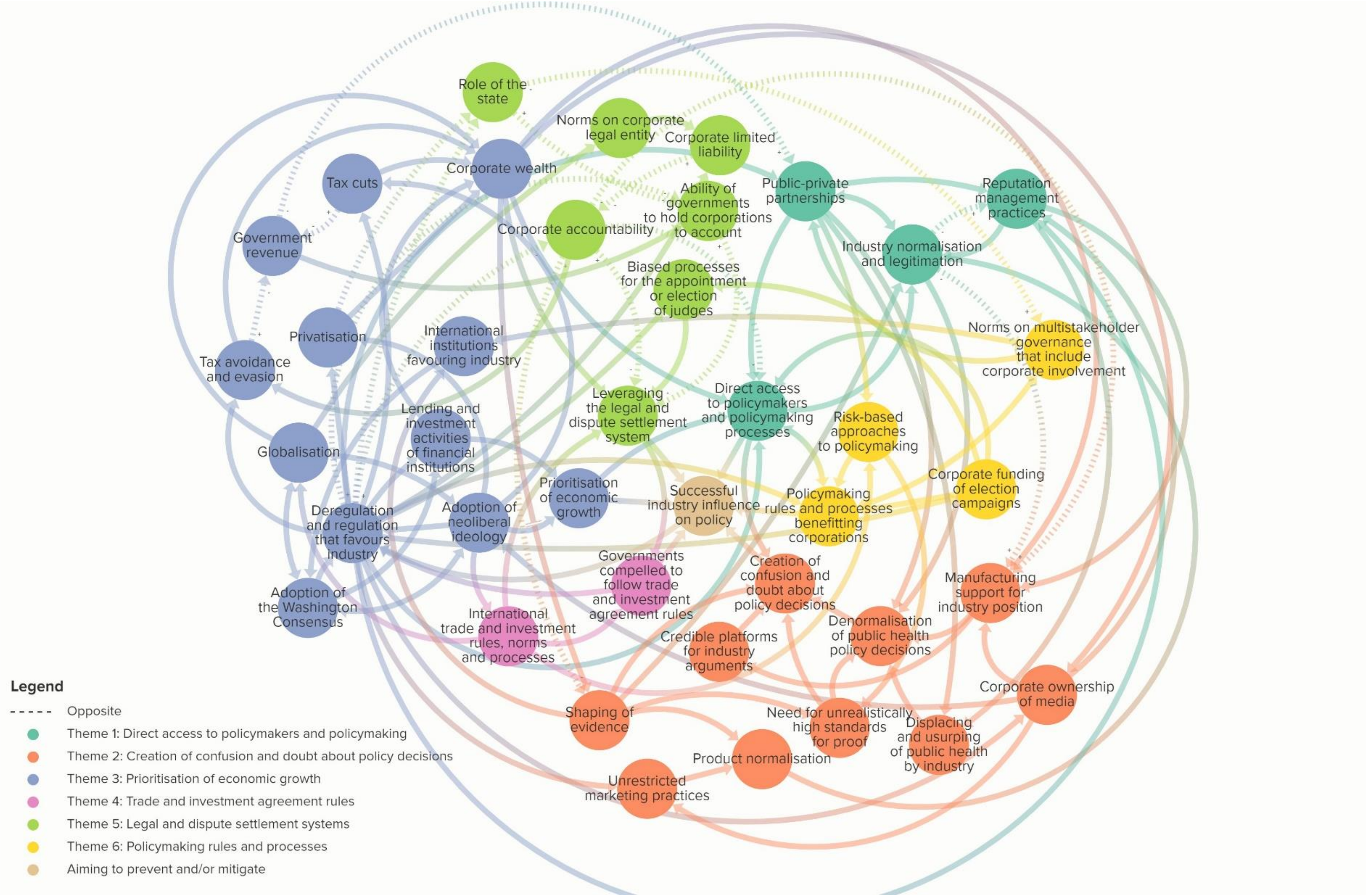

Preliminary systems map elements and theme changes

The preliminary systems map identified six themes consists of 40 elements. The following table shows the name of the preliminary map elements, themes and how they changed in relation to the final systems map. Element descriptions and interconnections for the final systems map is presented in supplementary file 3.

Preliminary system map element	Preliminary system map theme	Element change	Final system map element	Final system map theme
Ability of governments to hold corporations to account	Legal and dispute settlement systems (Theme 5)	Split	Ability of governments to hold corporations to account	Industry leveraging legal and dispute settlement processes (Theme 4)
			Governance capability	Direct access to public sector decision-makers (Theme 1)
Adoption of neoliberal ideology	Prioritisation of commercial growth (Theme 3)	Amended	Acceptance of neoliberalism	Corporate prioritisation of commercial profits and growth (Theme 3)
Adoption of the Washington Consensus	Prioritisation of commercial growth (Theme 3)	Same	Adoption of the Washington Consensus	Corporate prioritisation of commercial profits and growth (Theme 3)
Biased processes for the appointment or election of judges	Legal and dispute settlement systems (Theme 5)	Amended	Biased processes for the appointment or election of judicial officers or arbitrators	Industry leveraging legal and dispute settlement processes (Theme 4)
Corporate accountability	Legal and dispute settlement systems (Theme 5)	Integrated	Ability of governments to hold corporations to account	Industry leveraging legal and dispute settlement processes (Theme 4)
Corporate funding of election campaigns	Policymaking rules and processes (Theme 6)	Amended	Corporate funding of public officials' political activity	Industry leveraging policymaking, norms, rules, and processes (Theme 5)
Corporate limited liability	Legal and dispute settlement systems (Theme 5)	Same	Corporate limited liability	Industry leveraging legal and dispute settlement processes (Theme 4)
Corporate ownership of media	Creation of confusion and doubt about policy decisions (Theme 2)	Amended	Corporate control of media reporting	Creation of confusion and doubt about policy decisions (Theme 2)
Corporate wealth	Prioritisation of commercial growth (Theme 3)	Same	Corporate wealth	Corporate prioritisation of commercial profits and growth (Theme 3)
Creation of confusion and doubt about policy decisions	Creation of confusion and doubt about policy decisions (Theme 2)	Same	Creation of confusion and doubt about policy decisions	Creation of confusion and doubt about policy decisions (Theme 2)
Credible platforms for industry arguments	Creation of confusion and doubt about policy decisions (Theme 2)	Amended	Credible dissemination of industry arguments	Creation of confusion and doubt about policy decisions (Theme 2)
Denormalisation of public health policy decisions	Creation of confusion and doubt about policy decisions (Theme 2)	Amended	Denormalising public health policy	Creation of confusion and doubt about policy decisions (Theme 2)
Deregulation and regulation that favours industry	Prioritisation of commercial growth (Theme 3)	Amended	Implementation of UCI preferred laws, regulations, processes and norms	Outcome element
Direct access to policy makers and policymaking processes	Direct access to policymakers and policymaking (Theme 1)	Split and amended	Direct access to public sector decision-makers	Direct access to public sector decision-makers (Theme 1)
			Revolving doors	
Displacing and usurping of public health by industry	Creation of confusion and doubt (Theme 2)	Amended	Displacing and usurping of public health actors by industry	Creation of confusion and doubt about policy decisions (Theme 2)

Globalisation	Prioritisation of commercial growth (Theme 3)	Removed	--	--
Government revenue	Prioritisation of commercial growth (Theme 3)	Same	Government revenue	Corporate prioritisation of commercial profits and growth (Theme 3)
Governments compelled to follow trade and investment agreement rules	Trade and investment agreement rules (Theme 4)	Amended	Government obliged to follow international trade and investment agreements	Industry leveraging legal and dispute settlement processes (Theme 4)
Industry normalisation and legitimation	Direct access to policymakers and policymaking (Theme 1)	Same	Industry normalisation and legitimisation	Direct access to public sector decision-makers (Theme 1)
International institutions favouring industry	Prioritisation of commercial growth (Theme 3)	Amended	International business organisations favouring industry	Corporate prioritisation of commercial profits and growth (Theme 3)
International trade and investment rules, norms, and processes	Trade and investment agreement rules (Theme 4)	Amended	Competition law that favours corporations	Industry leveraging legal and dispute settlement processes (Theme 4)
Lending and investment activities of financial institutions	Prioritisation of commercial growth (Theme 3)	Same	Lending and investment activities of financial institutions	Corporate prioritisation of commercial profits and growth (Theme 3)
Leveraging the legal and dispute settlement system	Legal and dispute settlement systems (Theme 5)	Amended	Industry leveraging the legal and dispute settlement processes	Industry leveraging legal and dispute settlement processes (Theme 4)
Manufacturing support for industry position	Creation of confusion and doubt about policy decisions (Theme 2)	Amended	Generating support for industry position	Industry leveraging policymaking, norms, rules, and processes (Theme 5)
Need for unrealistically high standards of proof	Creation of confusion and doubt about policy decisions (Theme 2)	Amended	Contradictory standards of proof	Creation of confusion and doubt about policy decisions (Theme 2)
Norms on corporate legal entity	Legal and dispute settlement systems (Theme 5)	Amended	Corporate legal entity based on fiduciary duty	Industry leveraging legal and dispute settlement processes (Theme 4)
Norms on multistakeholder governance that include corporate involvement	Policymaking rules and processes (Theme 6)	Amended	Support for multistakeholder governance	Industry leveraging policymaking, norms, rules, and processes (Theme 5)
Policymaking rules and processes benefitting corporations	Policymaking rules and processes (Theme 6)	Amended	Industry leveraging policymaking, norms, rules, and processes	Industry leveraging policymaking, norms, rules, and processes (Theme 5)
Prioritisation of economic growth	Prioritisation of commercial growth (Theme 3)	Split	Government prioritisation of GDP growth<br>Corporate prioritisation of commercial profits and growth	Corporate prioritisation of commercial profits and growth (Theme 3)
Privatisation	Prioritisation of commercial growth (Theme 3)	Same	Privatisation	Corporate prioritisation of commercial profits and growth (Theme 3)
Product normalisations	Creation of confusion and doubt about policy decisions (Theme 2)	Amended	Product and brand normalisation	Creation of confusion and doubt about policy decisions (Theme 2)
Public-private partnerships	Direct access to policymakers and policymaking (Theme 1)	Same	Public-private partnerships	Direct access to public sector decision-makers (Theme 1)
Reputation management practices	Direct access to policymakers and policymaking (Theme 1)	Split	Corporate social responsibility practices<br>Reputation management practices	Direct access to public sector decision-makers (Theme 1)

Risk-based approaches to policymaking	Policymaking rules and processes (Theme 6)	Amended	Industry favoured regulatory approaches for policymaking	Industry leveraging policymaking, norms, rules, and processes (Theme 5)
Role of the state	Legal and dispute settlement systems (Theme 5)	Amended	Shrinking role of the state	Industry leveraging legal and dispute settlement processes (Theme 4)
Shaping of evidence	Creation of confusion and doubt about policy decisions (Theme 2)	Amended	Industry influence on evidence and science	Creation of confusion and doubt about policy decisions (Theme 2)
Successful industry influence on policy	Aiming to prevent and/or mitigate	Amended	UCI influence on health policy at local, national, or international levels	Target element
Tax avoidance and evasion	Prioritisation of commercial growth (Theme 3)	Split	Tax avoidance and evasion<br>Tax policy favouring corporations	Corporate prioritisation of commercial profits and growth (Theme 3)
Tax cuts	Prioritisation of commercial growth (Theme 3)	Integrated	Tax policy favouring corporations	Corporate prioritisation of commercial profits and growth (Theme 3)
Unrestricted marketing practices	Creation of confusion and doubt about policy decisions (Theme 2)	Amended	Marketing practices	Creation of confusion and doubt about policy decisions (Theme 2)
--	--	New	Cost of labour	Corporate prioritisation of commercial profits and growth (Theme 3)
--	--	New	Cultural legacy of industry	Creation of confusion and doubt about policy decisions (Theme 2)
--	--	New	Discontinuity of decision-makers	Creation of confusion and doubt (Theme 2)
--	--	New	Enmeshed social networks between representatives of public and private actors	Direct access to public sector decision-makers (Theme 1)
--	--	New	Externalities	Corporate prioritisation of commercial profits and growth (Theme 3)
--	--	New	Government corruption	Direct access to public sector decision-makers (Theme 1)
--	--	New	Government subsidies	Corporate prioritisation of commercial profits and growth (Theme 3)
--	--	New	Government use of consultancy firms	Creation of confusion and doubt about policy decisions (Theme 2)
--	--	New	Industry circumventing national departments of health	Policymaking norms, rules, and processes (Theme 5)
--	--	New	Institutional memory in public decision-making bodies	Creation of confusion and doubt about policy decisions (Theme 2)

--	--	New	International loan repayments	Corporate prioritisation of commercial profits and growth (Theme 3)
--	--	New	Market concentration of industry	Corporate prioritisation of commercial profits and growth (Theme 3)
		New	Monopolisation of supply chain	Corporate prioritisation of commercial profits and growth (Theme 3)
--	--	New	Perceived individual responsibility of unhealthy behaviours	Creation of confusion and doubt about policy decisions (Theme 2)
--	--	New	Policy Incoherence	Industry leveraging policymaking, norms, rules, and processes (Theme 5)
--	--	New	Politicians that support neoliberal policies gain votes in elections	Corporate prioritisation of commercial profits and growth (Theme 3)
--	--	New	Pricing strategies	Corporate prioritisation of commercial profits and growth (Theme 3)
--	--	New	Public health perspective of decision-makers	Corporate prioritisation of commercial profits and growth (Theme 3)
--	--	New	Regular change of government	Corporate prioritisation of commercial profits and growth (Theme 3)
--	--	New	Research literacy of decision-makers	Creation of confusion and doubt about policy decisions (Theme 2)
--	--	New	Shareholder primacy	Corporate prioritisation of commercial profits and growth (Theme 3)
--	--	New	Short-term view on policy decisions	Corporate prioritisation of commercial profits and growth (Theme 3)
