# Supplementary material for: Building a Systems Map: Applying Systems Thinking to Unhealthy Commodity Industry Influence on Public Health Policy
Source: Int J Health Policy Manag. 2024 Apr 7;13:7872. doi: 10.34172/ijhpm.2024.7872 (PMC11607592; doi:10.34172/ijhpm.2024.7872)
Supplement: Supplementary file 2 — Participant Knowledge Areas and Workshop Groups. [file ijhpm-13-7872-s002.pdf]

Article title: Building a Systems Map: Applying Systems Thinking to Unhealthy Commodity Industry Influence on Public Health Policy

Journal name: International Journal of Health Policy and Management (IJHPM)

Authors' information: Adam Bertscher[1]*, James Nobles[2], Anna B Gilmore[3], Krista Bondy[4], Amber van den Akker[3], Sarah Dance[5], Michael Bloomfield[1], Mateusz Zatoński[3]

[1]Department of Social and Policy Sciences, Faculty of Humanities & Social Sciences, University of Bath, Bath, UK.

[2]Centre of Active Lifestyles, Leeds Beckett University, Leeds, UK.

[3]Department for Health, Faculty of Humanities & Social Sciences, University of Bath, Bath, UK.

[4]School of Management, Marketing, Business & Society, University of Bath, Bath, UK.

[5]Department of Psychology, Faculty of Humanities & Social Sciences, University of Bath, Bath, UK.

Additional Information: Our dear co-author Mateusz Zatoński, PhD, sadly died on January 17, 2022.

***Correspondence to:** Adam Bertscher; Email: ab3298@bath.ac.uk

Citation: Bertscher A, Nobles J, Gilmore AB, et al. Building a systems map: applying systems thinking to unhealthy commodity industry influence on public health policy. Int J Health Policy Manag. 2024;13:7872. doi:10.34172/ijhpm.2024.7872

Supplementary file 2. Participant Knowledge Areas and Workshop Groups

Participant knowledge areas

Participant knowledge areas		Number of participants
**Stakeholder group**	Academia	26
	Civil society	19
	Former public official	4
	Global governance	2
**Expertise area***	Ultra-processed foods	27
	Alcohol	24
	Tobacco	29
	CDoH and/or industry influence	38
	Economics	1
	International trade	4
	Policymaking	12
	Law	5
**Geographical region of expertise* (WHO regions)**	African Region	17
	Region of the Americas	17
	South-East Asian Region	4
	European Region	27
	Eastern Mediterranean Region	1
	Western Pacific Region	15

* Participant expertise area (i.e., tobacco, alcohol etc) and geographical region of expertise (i.e., African region, European Region etc) may fall within more than one category
