# Supplementary material for: Building a Systems Map: Applying Systems Thinking to Unhealthy Commodity Industry Influence on Public Health Policy
Source: Int J Health Policy Manag. 2024 Apr 7;13:7872. doi: 10.34172/ijhpm.2024.7872 (PMC11607592; doi:10.34172/ijhpm.2024.7872)
Supplement: Supplementary file 3 — Element Descriptions and Interconnections. [file ijhpm-13-7872-s003.pdf]

Article title: Building a Systems Map: Applying Systems Thinking to Unhealthy Commodity Industry Influence on Public Health Policy

Journal name: International Journal of Health Policy and Management (IJHPM)

Authors' information: Adam Bertscher[1]*, James Nobles[2], Anna B Gilmore[3], Krista Bondy[4], Amber van den Akker[3], Sarah Dance[5], Michael Bloomfield[1], Mateusz Zatoński[3]

[1]Department of Social and Policy Sciences, Faculty of Humanities & Social Sciences, University of Bath, Bath, UK.

[2]Centre of Active Lifestyles, Leeds Beckett University, Leeds, UK.

[3]Department for Health, Faculty of Humanities & Social Sciences, University of Bath, Bath, UK.

[4]School of Management, Marketing, Business & Society, University of Bath, Bath, UK.

[5]Department of Psychology, Faculty of Humanities & Social Sciences, University of Bath, Bath, UK.

Additional Information: Our dear co-author Mateusz Zatoński, PhD, sadly died on January 17, 2022.

***Correspondence to:** Adam Bertscher; Email: ab3298@bath.ac.uk

Citation: Bertscher A, Nobles J, Gilmore AB, et al. Building a systems map: applying systems thinking to unhealthy commodity industry influence on public health policy. Int J Health Policy Manag. 2024;13:7872. doi:10.34172/ijhpm.2024.7872

Supplementary file 3. Element Descriptions and Interconnections

Element descriptions and interconnections

The final systems map consists of 64 elements across five interconnected themes.

Themes: Direct access to public sector decision-makers (Theme 1); Creation of confusion and doubt about policy decisions (Theme 2); Prioritisation of commercial profits and growth (Theme 3); Legal and dispute settlement processes (Theme 4); Policymaking, norms, rules, and processes (Theme 5)

Target element (i.e., elements that interventions would ultimately aim to decrease/reduce): UCI influence on health policy at local, national, or international levels (large red circle)

Outcome element (i.e., element that UCI influence produces): *UCI preferred outcomes* (large red rectangle)

Element	Theme	Description	Additional clarification	Interconnection
**Acceptance of neoliberalism**	Corporate prioritisation of commercial profits and growth (Theme 3)	The extent to which a governing body – at a local, subnational, national, regional, or international level – subscribes to neoliberalism.	Note:<br>- Neoliberalism is a "political approach and ideology often also referred to as ‘market fundamentalism’ or 'free market' ideology... [that] …emphasises private property rights and free markets as the way of organising human interaction, promotes privatisation, trade liberalisation, deregulation and reductions in tax and welfare payments with the role of the state pared back to ensuring the functioning of the market."[1]	Increase in the *acceptance of neoliberalism* enables or leads to an increase in:<br>- *Perceived individual responsibility of unhealthy behaviours* (Theme 2)<br>- *Denormalising public health policy* (Theme 2)<br>- *Government prioritisation of GDP growth* (Theme 3)<br>- *Tax policy favouring corporations* (Theme 3)<br>- *Privatisation* (Theme 3)<br>- *Corporate legal entity based on fiduciary duty* (Theme 3)<br>- *Adoption of the Washington Consensus* (Theme 3)<br>- *UCI preferred outcomes* (Outcome element)
**Adoption of the Washington Consensus**	Corporate prioritisation of commercial profits and growth (Theme 3)	The extent to which policy actors adopts the Washington Consensus (i.e., neoliberal policies) as international policy standards and best practice to achieve international development goals.	--	Increase in the *adoption of the Washington Consensus* enables or leads to an increase in:<br>- *Government prioritisation of GDP growth* (Theme 3)<br>- *Lending and investment activities of financial institutions* (Theme 3)<br>- *Acceptance of neoliberalism* (Theme 3)<br>- *UCI preferred outcomes* (Outcome element)
**Biased processes for the appointment or election of judicial officers or arbitrators**	Industry leveraging legal and dispute settlement processes (Theme 4)	The extent to which the appointment or election of judicial officers or arbitrators introduces risk of bias.	--	Increase in *biased processes for the appointment or election of judicial officers or arbitrators* enables or leads to increases in:<br>- *Industry leveraging the legal and dispute settlement processes* (Theme 4)
**Competition law that favours corporations**	Industry leveraging legal and dispute	The extent to which competition law, including	Note:	Increase *in competition law that favours corporations* enables or leads to increases in:

	settlement processes (Theme 4)	laws governing mergers and acquisitions and intellectual property law, favours corporations.	- This includes press laws that favour corporate concentration of media ownership.	- *Monopoly concentration of industry* (Theme 3)
**Contradictory standards of proof**	Creation of confusion and doubt about policy decisions (Theme 2)	The extent to which evidentiary standards of proof are applied inconsistently to industry and public health actors.	Explanation:<br>- The benchmark for standards of proof is particularly high for public health actors, which gives the perception that public health evidence is of poor quality. At the same time, industry demands high evidentiary standards, whilst their own research is of poor quality, and use questionable scientific methods to conduct their own research.	Increase in *contradictory standards of proof* enables or leads to increases in:<br>- *Creation of confusion and doubt about policy decisions* (Theme 2)<br>- *Denormalising public health policy* (Theme 2)<br>- *Displacing and usurping of public health actors by industry* (Theme 2)
**Corporate control of media reporting**	Creation of confusion and doubt about policy decisions (Theme 2)	The extent to which corporations can control what is reported in the media.	Assumptions:<br>- Advertising and media industries may be supportive of UCIs and oppose regulation as they rely on UCI marketing and/or sponsorships.<br>- As media industry ownership is consolidated under a few corporations, they have significant control over what is reported in the media. What is reported on tends to support neoliberal policies.<br>- Corporate control of reporting may lead to increase the possibility of politicians winning votes since the media tends to put a positive focus on political candidates that they favour.<br>- This element includes the use of novel technologies by the media, such as social media, to exert such influence.	Increase in *corporate control of media reporting* enables or leads to increases in:<br>- *Creation of confusion and doubt about policy decisions* (Theme 2)<br>- *Politicians winning votes in elections* (Theme 3)<br>- *Generating support for industry position* (Theme 5)<br>- *Reputation management practices* (Theme 5)<br>- *Contradictory standards of proof* (Theme 2)<br>- *Credible dissemination of industry arguments* (Theme 2)<br>- *Marketing practices* (Theme 3)<br>- *Acceptance of neoliberalism* (Theme 3)
**Corporate funding of public officials' political activity**	Direct access to public sector decision-makers (Theme 1)	The extent to which public officials, including their political parties, election campaigns or political action committees, are funded by corporations or one of their derived organisations (e.g., social aspects public relations organisation or industry associations).	--	Increase in *corporate funding of public officials' political activity* enables or leads to increases in:<br>- *Biased processes for the appointment or election of judicial officers or arbitrators* (Theme 4)<br>- *Government corruption* (Theme 1)<br>- *Direct access to public sector decision-makers* (Theme 1)

**Corporate legal entity based on fiduciary duty**	Corporate prioritisation of commercial profits and growth (Theme 3)	The extent to which the societal norm is accepted (codified into law) that the legal entity of a corporation should be based on its fiduciary duty to shareholders.	Note:<br>- The fiduciary duty of corporates may differ between countries depending on their domestic legislation.	Increase in the *corporate legal entity being based on fiduciary duty* enables or leads to an increase in:<br>- *Corporate limited liability* (Theme 4)<br>- *Shareholder primacy* (Theme 3)
**Corporate limited liability**	Industry leveraging legal and dispute settlement processes (Theme 4)	The extent to which the owners or management or a corporation or other entities are liable for the corporate debt, damages, or wrongdoing.	--	Increase in *corporate limited liability* enables or leads to increases in:<br>- *Industry leveraging the legal and dispute settlement processes* (Theme 4)<br><br>Increase in *corporate limited liability* leads to a decrease in:<br>*Ability of governments to hold corporations to account* (Theme 4)
**Corporate prioritisation of commercial profits and growth**	Corporate prioritisation of commercial profits and growth (Theme 3)	The extent to which UCIs prioritise their own commercial growth above societal goals and values, such as health, well-being, human rights, or the natural environment.	--	Increase in the *Corporate prioritisation of commercial profits and growth* enables or leads to an increase in:<br>- *Industry influence on public health policy at local, national, or international levels* (Target element)<br>- *Industry leveraging policymaking, norms, rules, and processes* (Theme 5)<br>- *Creation of confusion and doubt about policy decisions* (Theme 2)<br>- *Policy incoherence* (Theme 5)<br>- *Monopoly concentration of industry* (Theme 3)<br>- *Externalities* (Theme 3)
**Corporate social responsibility practices**	Direct access to public sector decision-makers (Theme 1)	The extent to which corporations promote and deploy their corporate social responsibility activities. Corporate social responsibility is the voluntary commitment corporations make to "hold ethical norms and refrain from causing harm".[1]	Examples:<br>- Sustainability and corporate citizenship, often in the form of self-regulation<br>- Participating in prosocial activities, such as public-private partnerships, promoting harm reduction, or charitable donations	Increase in *corporate social responsibility practices* enables or leads to increases in:<br>- *Generating support for industry position* (Theme 5)<br>- *Public-private partnerships* (Theme 1)<br>- *Reputation management practices* (Theme 1)<br>- *Product and brand normalisation* (Theme 2)<br>- *Displacing and usurping of public health actors by industry* (Theme 2)<br>- *Industry normalisation and legitimisation* (Theme 1)
**Corporate wealth**	Corporate prioritisation of commercial profits and growth (Theme 3)	The number of financial resources (i.e., money and assets) a corporation owns.	Note:<br>- Different sized corporations may have different impacts on policy. Some individual corporations are so large and powerful in terms of money and resources as to have disproportionate influence. Their motivation may differ from that of smaller companies, such as concerns about regulatory diffusion	Increase *corporate wealth* enables or leads to an increase in:<br>- *Generating support for industry position* (Theme 5)<br>- *Government corruption* (Theme 1)<br>- *Direct access to public sector decision-makers* (Theme 1)<br>- *Industry leveraging policymaking, norms, rules, and processes* (Theme 5)<br>- *Corporate control of media industry* (Theme 2)<br>- *Industry leveraging the legal and dispute settlement processes* (Theme 4)

			between local, national, and international governance organisations.	- *Industry influence on evidence and science* (Theme 2)<br>- *Displacing and usurping of public health actors by industry* (Theme 2)<br>- *Revolving doors* (Theme 1)<br>- *Corporate funding of a public official's political activity* (Theme 1)<br>- *Public-private partnerships* (Theme 1)
**Creation of confusion and doubt about policy decisions**	Creation of confusion and doubt about policy decisions (Theme 2)	The extent to which decision-makers, and the public are confused about or doubtful that the proposed policy will lead to positive or desirable public value.	Note:<br>- Industry uses various arguments to undermine policy to created confusion and doubt policy decisions. A full taxonomy of such arguments is outlined by Ulucanlar et al, 2023	Increase in *creation of confusion and doubt about policy decisions* enables or leads to increases in:<br>- *Industry influence on health policy at local, national, or international levels* (Target element)<br>- *Industry leveraging the legal and dispute settlement processes* (Theme 4)
**Credible dissemination of industry arguments**	Creation of confusion and doubt about policy decisions (Theme 2)	The extent to which industry creates a perception of producing credible information, disseminated through networks of actors.	Examples:<br>- "Use legitimate scientific platforms to showcase industry-sponsored research, using independent scientists."[2]<br>- "Cite industry-supporting and industry-produced studies widely in (peer-reviewed) journal articles and other information sources." [2]<br>- "Widely disseminate favourable research and information using a variety of media and input from business and civil society allies." [2]	Increase in the *credible dissemination of industry arguments* leads to an increase in:<br>- *Creation of confusion and doubt about policy decisions* (Theme 2)<br>- *Generating support for industry position (*Theme 5)
**Cultural legacy of industry**	Creation of confusion and doubt about policy decisions (Theme 2)	The extent to which an industry is perceived to have historical cultural significance for the country or society.	--	Increase in *cultural legacy of industry* leads to an increase in:<br>- *Product and brand normalisation* (Theme 2)<br>- *Industry normalisation and legitimisation* (Theme 1)
**Denormalising public health policy**	Creation of confusion and doubt about policy decisions (Theme 2)	The extent to which industry creates the perception that public health policy interventions, are unnecessary or socially unacceptable (i.e., not preferred, or undesirable policy options).	--	Increase in the *denormalisation of public health policy* leads to an increase in:<br>- *Creation of confusion and doubt about policy decisions* (Theme 2)<br>- *Generating support for industry position* (Theme 5)
**Direct access to public sector decision-makers**	Direct access to public sector decision-makers (Theme 1)	The extent to which public sector decision-makers (policymakers, civil servants, public officials) have been directly accessed by industry actors through different types	Examples:<br>- The use of incentives (gifts) & threats (disinvestment) to policymakers. [2]<br>- Acquire roles within policymaking bodies (sitting on advisory boards). [2]	Increase in *direct access to public sector decision-makers* enables or leads to increases in:<br>- *Industry influence on public health policy at local, national, or international levels* (Target element).<br>- *Creation of confusion and doubt about policy decisions* (Theme 2)

		of formal and informal relationships or links to industry representatives.	- Industry "gifts" data so governments feel obligated to let industry into policymaking processes.<br>- Industry requesting meetings with public officials so often that it encroaches on their decision-making space and risks preventing public officials from developing policy.	- *Industry leveraging policymaking, norms, rules, and processes* (Theme 2)<br>- *Industry normalisation and legitimisation* (Theme 1)
**Discontinuity of decision-makers**	Creation of confusion and doubt about policy decisions (Theme 2)	The extent to which the public officials remain in their positions as decision-makers over time.	--	Increase in *continuity of decision-makers* leads to decrease in:<br>- *Institutional memory in public decision-making bodies* (Theme 2)
**Displacing and usurping of public health actors by industry**[3]	Creation of confusion and doubt about policy decisions (Theme 2)	The extent to which industry attempts to marginalise and take over the role of public health actors.	Examples:<br>- Offering alternative forms of regulation that are ineffective (distraction policies or fig leaf policies)[2]<br>- "Initiate self-regulation and voluntary codes relating to, for example, labelling, 'conscious' advertising, 'responsible' marketing, reformulation, etc., to prevent binding regulation."[2]<br>- "Contribute to funding, planning, delivery and evaluation of life-style information and education interventions that also emphasize responsible consumption"[2]<br>- "Deliver interventions that do not impact on the sale of products (e.g. in obesity, interventions designed to encourage exercise instead of changing consumption of food)."[2]<br>- Develop and market "narrow the focus of interventions and market 'healthier' versions of products (e.g. Diet Coke, e-cigarettes or low-alcohol drink), nutraceuticals, etc, as substitutes."[2]<br>- "Produce educational materials, books, guidelines and organise workshops for health professionals in partnership with civil society, patient and professional organisations, government	Increase in the *displacing and usurping of public health actors by industry* leads to an increase in:<br>- *Denormalising public health policy* (Theme 2)

			departments and global organisations."[2]<br>- "Infiltrate/monitor public health advocacy groups and independent researchers, threaten to withdraw support, intimidate individuals." [2]<br>- "Defame public health researchers, advocates and organisations through attacks on their work and personal integrity"[2]<br>- Undermine the capacity of public health institutions to respond to the public interests[2]	
**Enmeshed social networks between representatives of public and private actors**	Direct access to public sector decision-makers (Theme 1)	The extent to which public sector decision-makers' social networks are intertwined with senior corporate management.	Note:<br>- Past relationships through an individual's social networks that allow connections to be made between representatives from the public and private sectors, such as through family connections or attending the same schools, colleges, or universities.	Increase in *enmeshed social networks between representatives of public and private actors* enables or leads to increases in:<br>- *Direct access to public sector decision-makers* (Theme 1)<br>- *Public-private partnerships* (Theme 1)<br>- *Revolving doors* (Theme 1)
**Externalities**	Corporate prioritisation of commercial profits and growth (Theme 3)	The extent to which costs of the production and consumption of an unhealthy commodity is involuntarily incurred by a third party.	Example:<br>- The cost of smoking is incurred by treating patients in the public health system or is incurred by those in proximity to the smoker being exposed to second harm smoke.<br>- The costs of alcohol-related harms are incurred not only by the health system but by communities due to the subsequent crime and violence.	Increase in *externalities* enables or leads to an increase in:<br>- *Corporate wealth* (Theme 3)<br>- *Lending and investment activities of financial institutions* (Theme 3)<br><br>Increase in *externalities* leads to a decrease in:<br>- Government revenue (Theme 3)
**Generating support for industry position**	Industry leveraging policymaking, norms, rules, and processes (Theme 5)	The extent to which corporate actors fabricate or galvanise support with stakeholders.	Examples:<br>- Forming alliances with within industry[2]<br>- Recruit other industries as allies (e.g., advertising industry) [2]<br>- Forming alliances with key individuals and pre-existing organisations[2]<br>- Fabricate allies by setting up front groups, fake grassroots organisations, social aspect public relations organisations and think-tanks. [2]	Increase in *generating support for industry position* enables or leads to an increase in:<br>- *Credible dissemination of industry arguments* (Theme 2)<br>- *Direct access to public sector decision-makers* (Theme 1)<br>- *Denormalising public health policy* (Theme 2)<br>- *Industry leveraging policymaking, norms, rules, and processes* (Theme 5)

			- “Direct all forms of allies to engage in campaign activities including recruiting other allies, producing and disseminating information, media advocacy, responding to policy consultations, lobbying, initiating legal action and agitating on behalf of industry”.[2]<br>- Industry allies “hide links between allies… and industry to render their messages, actions and evidence more credible and acceptable.” [2]<br>- Paying journalists or researchers to write or speak about issues.[2]	
**Governance capability**	Direct access to public sector decision-makers (Theme 1)	The extent to which governments have the ability achieve their intended goals.	Note:<br>- This is impacted by the government funding, resources, and numbers of staff and their competencies that allow governments to operate effectively.	Increase in the *governance capability* enables or leads to an increase in:<br>- *Ability of governments and civil society to hold corporations to account* (Theme 4)<br><br>Increase in *governance capability* disables or leads to a decrease in:<br>- *Public-private partnerships* (Theme 1)<br><br>Decrease in *governance capability* disables or leads to an increase in:<br>- *Government corruption* (Theme 1)<br><br>Decrease in *governance capability* enables or leads to an increase in:<br>- *Industry circumventing national departments of health* (Theme 5)
**Government corruption**	Direct access to public sector decision-makers (Theme 1)	The extent to which extra-legal (but unethical) or illegal corruption (i.e., abuse of entrusted power for private gain) is endemic in a government.	Assumption:<br>- Corruption usually results in a conflict of interests for decision-makers when making policy<br><br>Examples:<br>- Bribes to public officials<br>- Extortion<br>- Industry spying<br>- Revolving doors	Increase in *government corruption* enables or leads to increases in:<br>- *Direct access to public sector decision-makers* (Theme 1)<br>- *Industry leveraging policymaking, norms, rules, and processes* (Theme 5)<br>- *Biased processes for the appointment or election of judicial officers or arbitrators* (Theme 4)

**Government obliged to follow international trade and investment agreements**	Industry leveraging legal and dispute settlement processes (Theme 4)	The extent to which governments are obligated to adhere to international trade and investments agreements.	Note:<br>- By entering into international trade and investment agreement, countries may be required to conduct risk assessments. This has resulted in a proliferation the use of these tools in LMICS.<br>- There may be safeguards for public health in international trade and investment agreements, such as minimum unit pricing in Scotland, which was judged to be compatible with EU law. However, such agreements can still create regulatory chill (i.e., legal uncertainty and procedurally industry can use them to delay policy development and implementation)	Increase in *government obligations to follow international trade and investment agreements* enables or leads to increases in:<br>- *Industry leveraging the legal and dispute settlement processes* (Theme 4)<br>- *Policy incoherence* (Theme 5)<br>- *Corporate prioritisation of commercial profits and growth* (Theme 3)<br>- *Lending and investment activities of financial institutions* (Theme 3)<br>- *Tax avoidance and evasion* (Theme 3)<br>- *Tax policy favouring corporations* (Theme 3)<br>- *Industry favoured regulatory approaches for policymaking* (Theme 5)<br>- *Competition law that favours corporations* (Theme 4)
**Government prioritisation of GDP growth**	Corporate prioritisation of commercial profits and growth (Theme 3)	The extent to which society prioritises the notion of GDP growth as a measure of national and global economic growth, without taking into full consideration full economic costs or societal values, such as health, well-being, human rights, or the natural environment.	Note:<br>- There may often be an economic case for particular public health interventions. However, governments usually give more priority to short-term approaches that tend to increase economic growth and job creation.	Increase in the *government prioritisation of GDP growth* enables or leads to an increase in:<br>- *Short-term view on policy decisions* (Theme 3)<br>- *Corporate prioritisation of commercial profits and growth* (Theme 3)<br>- *Policy incoherence* (Theme 5)
**Government revenue**	Corporate prioritisation of commercial profits and growth (Theme 3)	The amount of government revenue received.	Note:<br>- Government revenue can be received through taxes or loan repayments or can decrease by repaying loan.	Increase in *government revenue* or leads to an increase in:<br>- *Ability of governments to hold corporations to account* (Theme 4)<br>- *Governance capability* (Theme 1)<br><br>Decrease in *government revenue* enables or leads to an increase in:<br>- *Public-private partnerships* (Theme 1)
**Government subsidies**	Corporate prioritisation of commercial profits and growth (Theme 3)	The extent to which UCIs receive government subsidies.	--	Increase in *government subsidies* enables or leads to an increase in:<br>- *Corporate wealth* (Theme 3)<br><br>Increase in *government subsidies* enables or leads to a decrease in:<br>- *Government revenue* (Theme 3)

**Government use of consultancy firms**	Creation of confusion and doubt about policy decisions (Theme 2)	The extent to which governments employ consultancy firms to help make policy decisions.	Note:<br>- Governments departments commission consultancies to conduct research to help make policy recommendations. These recommendations are based on a narrow set of beliefs, assumptions, and expectations (such as neoliberalism) and may favour industry rather than public health goals.	Increase in *government use of consultancy firms* enables or leads to an increase in:<br>- *Tax policy favouring corporations* (Theme 3)<br>- *Privatisation* (Theme 3)<br>- *Creation of confusion and doubt about policy decisions* (Theme 2)<br>- *Perceived individual responsibility of unhealthy behaviours* (Theme 2)
**Industry circumventing national departments of health**	Industry leveraging policymaking, norms, rules, and processes (Theme 5)	The extent to which industry circumvents national departments of health.	Note:<br>- Industry may circumvent national departments of health by galvanising support from other supportive departments, for example, the treasury.	Increase in *industry circumventing national departments of health* enables or leads to an increase in:<br>- *Industry leveraging policymaking, norms, rules, and processes* (Theme 5)
**Industry favoured regulatory approaches for policymaking**	Industry leveraging policymaking, norms, rules, and processes (Theme 5)	The extent to which governments establish regulatory approaches that mandate industry favoured policymaking procedures, such as impact assessments, stakeholder consultations, and risk assessments	Note:<br>- The requirements for government departments to conduct impact assessments, stakeholder consultations, and risk assessments can all be leveraged by industry in their favour, which has been seen in "Better Regulation" in the UK and EU.<br>- These regulatory approaches include the extent to which the norm is accepted that policy should be made to mitigate a certain level of risk (which is challenging to reach using scientific methods) instead of creating policies using the precautionary principle that aims to avoid a possibility of harms. Risk-based approaches set a high evidentiary standard where a product may need to have a high possibility of risk before it can be regulated, which may be challenging to prove.	Increase in *industry favoured regulatory approaches for policymaking* enables or leads to an increase in:<br>- *Industry leveraging policymaking, norms, rules, and processes* (Theme 5)<br>- *Contradictory standards of proof* (Theme 2)
**Industry influence on evidence and science**[3]	Creation of confusion and doubt about policy decisions (Theme 2)	The extent to which industry funds, produces, controls, and manages information and research.	Examples:<br>- "Criticise (independent) research unfavourable to industry using unachievable evidentiary standards and non-rigorous methods."[2]<br>- "Misreport, 'cherry-pick' and misinterpret research and information unfavourable to industry."[2]	Increase in the *industry influence on evidence and science* enables or leads to increases in:<br>- *Creation of confusion and doubt about policy decisions* (Theme 2)<br>- *Product and brand normalisation* (Theme 2)<br>- *Industry normalisation and legitimisation* (Theme 1)<br>- *Displacing and usurping of public health actors by industry* (Theme 2)

			- "Over-emphasize complexity, uncertainty and disagreement among researchers."[2]<br>- Hide unfavourable evidence produced or funded by industry[2]<br>- "Produce or commission external institutions and scientists to create a self-referential body of alternative research that contradicts the international research literature." [2]<br>- "Cite inaccessible, unpublished, unverifiable or non-peer-reviewed evidence."[2]	- *Credible dissemination of industry arguments* (Theme 2)<br>- *Denormalising public health policy* (Theme 2)<br>- *Perceived individual responsibility of unhealthy behaviours* (Theme 2)<br>- *Industry leveraging the legal and dispute settlement processes* (Theme 4)<br>- *Public-private partnerships* (Theme 1)<br>- *Generating support for industry position* (Theme 5)<br>- *Contradictory standards of proof* (Theme 2)
**UCI influence on public health policy at local, national, or international levels**	Target element (large red circle)	The extent to which industry suppresses, shapes, weakens, blocks, or delays policy at the subnational, national, regional, supranational, or international levels. This includes all stages of the policy cycle (i.e., agenda setting, formulation, implementation, and evaluation).	Note:<br>- This is the main element of interest; the five elements leading to it represents the pathways through which industry influences policy.<br><br>Assumption:<br>- If these pathways were disrupted or minimised in equal measure, then UCIs may be reduced. If only one or a few pathways are blocked, then industry may adapt to exert influence through remaining pathways.	Increase in Industry influence on public health policy at local, national, or international levels leads to increases in:<br>- *UCI preferred outcomes* (Outcome element)
**Industry leveraging policymaking, norms, rules, and processes**	Industry leveraging policymaking, norms, rules, and processes (Theme 5)	The extent to which industry leverages national and international policymaking norms, rules and processes that favour their participation.	Example:<br>- Industry participates in policy consultations through mandatory regulatory impact assessments, requirements to involve industry, or processes for private and public sectors to work together to develop public policy (e.g., UK's Responsibility Deal)<br><br>Note:<br>- The disparity in resources between public and private sector actors allows for industry to use their resources to request meetings so often that it risks preventing public officials from develop policy.	Increase in *industry leveraging policymaking rules and processes* enables or leads to an increase in:<br>- *Industry influence on public health policy at local, national, or international levels* (Target element)<br>- *Direct access to public sector decision-makers* (Theme 1)<br>- *Creation of confusion and doubt about policy decisions* (Theme 2)

			- Usually, government rules do not require an impact assessment for self-regulation or voluntary agreements, even when proposed explicitly as an alternative to regulation.	
**Industry leveraging the legal and dispute settlement processes**	Industry leveraging legal and dispute settlement processes (Theme 4)	The extent to which industry leverages legal and dispute settlement processes to produce industry favoured policy outcomes.	Example:<br>- Industry litigates or threatens to litigate against governments to prevent, undermine or reverse policy (leading to "regulatory chill").	Increase in *industry leveraging the legal and dispute settlement processes* enables or leads to increases in:<br>- *Industry influence on public health policy at local, national, or international levels* (Target element)
**Industry normalisation and legitimisation**	Direct access to public sector decision-makers (Theme 1)	The extent to which an industry is seen as socially acceptable (credible and desirable) and thus a legitimate policy actor.	Note<br>- The tobacco industry is denormalised to a certain extent, especially compared to the food and alcohol industries.	Increase in *industry normalisation and legitimisation* enables or leads to increases in:<br>- *Direct access to public sector decision-makers* (Theme 1)<br>- *Industry influence on evidence and science* (Theme 2)<br>- *Product and brand normalisation* (Theme 2)<br>- *Denormalising public health policy* (Theme 2)<br>- *Public-private partnerships* (Theme 1)<br><br>Decreased in *industry being normalised and legitimised* enables or leads to increases in:<br>- *Corporate social responsibility practices* (Theme 1)<br>- *Generating support for industry position* (Theme 5)
**Institutional memory in public decision-making bodies**	Creation of confusion and doubt about policy decisions (Theme 2)	The extent to which a decision-makers representing a public institution, such as government departments or other decision-making bodies, share past experiences and knowledge of public health issues over time.	Assumption:<br>- If decision-makers are in office for longer periods of time, they can build up expertise and understanding about public health issues and implement long term policy strategies.	Increase in the *institutional memory in public decision-making bodies* leads to an increase in:<br>- *Research literacy of decision-makers* (Theme 2)<br><br>Increase in the *institutional memory in public decision-making bodies* leads to a decrease in:<br>- *Creation of confusion and doubt about policy decisions* (Theme 2)
**International business organisations favouring industry**	Corporate prioritisation of commercial profits and growth (Theme 3)	The extent to which international organisations representing business, such as World Economic Forum, the World Business Council for Sustainable Development and the International Chamber of Commerce promote neoliberal policies and the role of the private sector in governance.	Note:<br>- International business organisations may not necessarily have direct influence on policy making, but they may contribute to a "climate of opinion", through their support of multistakeholder governance, support for neoliberal policies and supporting industry regulatory approaches, such as self-regulation.	Increase in *international business organisations favouring industry* leads to an increase in:<br>- *Support for multistakeholder governance* (Theme 5)<br>- *Acceptance of neoliberalism* (Theme 3)<br>- *Displacing and usurping of public health actors by industry* (Theme 2)

**International loan repayments**	Corporate prioritisation of commercial profits and growth (Theme 3)	The amount of money being either received or being paid for loan repayments.	Note:<br>- This element depends on which government is repaying the loan and which is receiving the loan repayment. This element can decrease revenue in countries where they are repaying the loan or increase revenue of governments that receive the loan repayment.	Increase in *international loan repayments* enables or leads to an increase in:<br>- *Government revenue* (Theme 3)<br><br>Increase in *international loan repayments* disables or leads to a decrease in:<br>- *Government revenue* (Theme 3)
**Cost of labour**	Corporate prioritisation of commercial profits and growth (Theme 3)	The cost of labour	Assumption<br>- If labour costs are kept low this can increase corporate wealth.	Increase in labour costs enables or leads to a decrease in:<br>- *Corporate wealth* (Theme 3)
**Lending and investment activities of financial institutions**	Corporate prioritisation of commercial profits and growth (Theme 3)	The extent to which lending and investment practices of public and private financial institutions, such as commercial banks, investment banks, insurance companies, and brokerage firms, provide capital for UCIs to grow their business.	--	Increase in *lending and investment activities of financial institutions* leads to an increase in:<br>- *Corporate prioritisation of commercial profits and growth* (Theme 3)<br>- *Government prioritisation of GDP growth* (Theme 3)<br>- *International loan repayments* (Theme 3)<br>- *Corporate wealth* (Theme 3)<br>- *Government revenue* (Theme 3)
**Marketing practices**	Creation of confusion and doubt about policy decisions (Theme 2)	The extent to which corporate marketing practices are unrestricted or poorly monitored and enforced.	--	Increase in *marketing practices* enables or leads to increases in:<br>- *Corporate control of media industry* (Theme 2)<br>- *Corporate wealth* (Theme 3)<br>- *Product and brand normalisation* (Theme 2)<br>- *Corporate social responsibility practices* (Theme 1)
**Market concentration of industry**	Corporate prioritisation of commercial profits and growth (Theme 3)	The extent to which UCIs are concentrated through monopolies.	Assumption<br>- The wealth and power of corporations become so unequalled that it is beyond the capabilities of civil society and government to hold them to account.	Increase in the *monopoly concentration of industry* leads to an increase in:<br>- *Corporate wealth* (Theme 3)<br>- *Ability of governments to hold corporations to account* (Theme 4)<br>- *Corporate control of media reporting* (Theme 2)<br>- *Monopolisation of supply chains* (Theme 3)
**Monopolisation of supply chain**	Corporate prioritisation of commercial profits and growth (Theme 3)	The extent to which corporations monopolise their supply chain.	--	Increase in the m*onopolisation of supply chain* leads to an increase in:<br>- *Pricing strategies* (Theme 3)<br>- *Cost of labour* (Theme 3)
**UCI preferred outcomes**	Outcome element (large red rectangle)	The extent to which UCIs achieve their preferred outcomes, such as national and	Note:<br>- This element leads to many other elements in the systems map, which	Increase in *UCI preferred outcomes* enables or leads to increases in:<br>- *Tax avoidance and evasion* (Theme 3)

		international regulatory approaches, regulations, policies, laws, and norms that favour or benefit them.	creates feedback that reinforces and perpetuate UCI influence on policy.<br>- This element includes both public health policy (policies that aims to restrict marketing, availability, and affordability of, and access to, unhealthy commodity) and policy architecture (laws, regulations, processes and norms) in which policy is made.	- *Privatisation* (Theme 3)<br>- *Pricing strategies* (Theme 3)<br>- *Externalities* (Theme 3)<br>- *Government subsidies* (Theme 3)<br>- *Government obliged to follow international trade and investment agreements* (Theme 5)<br>- *Tax policy favouring corporations* (Theme 3)<br>- *Lending and investment activities of financial institutions* (Theme 3)<br>- *Corporate legal entity based on fiduciary duty* (Theme 4)<br>- *Industry leveraging policymaking, norms, rules, and processes* (Theme 5)<br>- *Corporate limited liability* (Theme 4)<br>- *Direct access to public sector decision-makers* (Theme 1)<br>- *Public-private partnerships* (Theme 1)<br>- *Corporate funding of a public official's political activity* (Theme 1)<br>- *Corporate social responsibility practices* (Theme 1)<br>- *Competition law that favours corporation* (Theme 5)<br>- *Revolving doors* (Theme 1)<br>- *Marketing practices* (Theme 2)<br>- *Support for multistakeholder governance* (Theme 5)<br>- *Biased processes for the appointment or election of judicial officers or arbitrators* (Theme 4)<br>- *Industry favoured regulatory approaches for policymaking* (Theme 5)<br>- Tax policy favouring corporations (Theme 3)<br><br>Increase in *UCI preferred outcomes* enables or leads to decreases in:<br>- *Shrinking role of the state* (Theme 4)<br>- *Ability of governments to hold corporations to account* (Theme 4)<br>- *Cost of labour* (Theme 3)
**Perceived individual responsibility of unhealthy behaviours**	Creation of confusion and doubt about policy decisions (Theme 2)	The extent to which extent to which individuals are perceived as being responsible for their own patterns of consumption behaviours.	--	Increase in *perceived individual responsibility of unhealthy behaviours* enables or leads to increases in:<br>- *Creation of confusion and doubt about policy decisions* (Theme 2)<br>- *Denormalising public health policy* (Theme 2)
**Policy Incoherence**	Industry leveraging policymaking, norms,	The extent to which there is inconsistent policy goals between government	Example:<br>- A department for economic development or trade has a mandate to	Increase in *policy incoherence* enables or leads to an increase in:

	rules, and processes (Theme 5)	departments. Government departments may seek to satisfy their own policy goals that may be contrary to the goals of other government departments.	increase trade and overall economic growth through increased consumption of goods, whereas a department for health has a mandate to decrease the consumption of unhealthy products.	- *Industry circumventing national departments of health* (Theme 5)
**Pricing strategies**	Corporate prioritisation of commercial profits and growth (Theme 3)	The extent to which UCIs apply to increase sales,	--	Increase in *pricing strategies* to an increase in:<br>- *Corporate wealth* (Theme 3)
**Politicians that support neoliberal policies gain votes in elections**	Corporate prioritisation of commercial profits and growth (Theme 3)	The extent to which politicians that support neoliberal policies gain votes in elections.	Assumption:<br>- This applies to states that have elections.<br>- Making election promises, such as increasing economic growth and job creation, are likely to attract more votes.	Increase in *politicians seeking to win votes in elections* enables or leads to increases in:<br>- *Short-term view on policy decisions* (Theme 3)<br>- *Regular change of government* (Theme 3)
**Privatisation**	Corporate prioritisation of commercial profits and growth (Theme 3)	The extent to which there is a shift in the provisions of services or products that were previously provided by the public sectors are instead provided by the private sector.	--	Increase in *privatisation* enables or leads to an increase in:<br>- *Corporate wealth* (Theme 3)<br>- *Shrinking role of the state* (Theme 4)
**Product and brand normalisation**	Creation of confusion and doubt about policy decisions (Theme 2)	The extent to which the product being consumed is seen as socially acceptable and – at times – especially desirable.	Note:<br>- Tobacco products and brands are denormalised to a certain extent, compared to food and alcohol products and brands. At the same time, some products and brands are seen as especially desirable, such as certain alcoholic beverages.<br>- Product and brand normalisation can lead to increased consumption of certain products and brands, which in turn leads to increased profits and thus corporate wealth.	Increase in *product and brand normalisation* enables or leads to increases in:<br>- *Corporate social responsibility practices* (Theme 1)<br>- *Industry normalisation and legitimisation* (Theme 1)<br>- *Corporate wealth* (Theme 3)<br>- *Creation of confusion and doubt about policy decisions* (Theme 2)
**Public health perspective of decision-makers**	Corporate prioritisation of commercial profits and growth (Theme 3)	The extent to which decision-makers adopts a public health lens in making policy decisions.	Assumption:<br>- The education and training of decision-makers, such as economics or law, inculcates them with a particular way of thinking about different policy decisions, making them less familiar with public health policy decisions and	Increase in *public health perspective of decision-makers* disables or may lead to a decrease in:<br>- *Government prioritisation of GDP growth* (Theme 3)<br>- *Acceptance of neoliberalism* (Theme 3)<br>- *Government obliged to follow international trade and investment agreements* (Theme 4)

			more focused on neoliberal policies decisions.	
**Public-private partnerships**	Direct access to public sector decision-makers (Theme 1)	The extent to which public and private sector actors at the subnational, national, regional, or international levels enter into partnerships to achieve a 'public good'.	Note:<br>- Industry sometimes interpreted "partnership" as meaning they are in one team with government with no distinction of role.<br>- Public-private partnerships may result in conflict of interest when making policy decisions.<br><br>Examples:<br>- Corporate funding of public or private actors<br>- The provision of welfare or public services by private actors<br>- Academic institutions collaborating with industry actors	Increase in *public-private partnerships* enables or leads to increases in:<br>- *Support for multistakeholder governance* (Theme 5)<br>- *Industry favoured regulatory approaches for policymaking* (Theme 5)<br>- *Direct access to public sector decision-makers* (Theme 1)<br>- *Industry normalisation and legitimisation* (Theme 1) "<br>- *Denormalising public health policy* (Theme 2)<br>- *Corporate social responsibility practices* (Theme 1)<br>- *Industry influence on evidence and science* (Theme 2)<br>- *Displacing and usurping of public health actors by industry* (Theme 2)<br>- *International business organisations favouring industry* (Theme 3)
**Regular change of government**	Corporate prioritisation of commercial profits and growth (Theme 3)	The extent to which there is regular change of government.	Assumptions:<br>- Governments may change for various reasons, such as regular democratic elections or coup d'états etc.<br>- If government do not have regular democracy elections, there may be seldom change of government.	Increase in *regular change of government* enables or leads to an increase in:<br>- *Short-term view on policy decisions* (Theme 3)<br>- *Discontinuity of decision-makers* (Theme 2)
**Reputation management practices**	Direct access to public sector decision-makers (Theme 1)	The extent to which corporations deploy practices to enhance their corporate brand image. [1]	Examples:<br>- Publicly associate with respected individuals and organisations[2]<br>- Use of different media to demonstrate positive commercial behaviour<br>- Working with competitors to create industry bodies to improve positive image and reduce the impact of negative incidents (e.g., oil spills) on individual organisations and thus the industry.<br>- Use of public relation companies to develop an image/branding strategy[1]	Increase in *reputation management practices* enables or leads to increases in:<br>- *Product and brand normalisation* (Theme 2)<br>- *Industry normalisation and legitimisation* (Theme 1)<br>- *Corporate social responsibility practices* (Theme 1)<br>- *Cultural legacy of industry* (Theme 2)<br>- *Corporate control of media reporting* (Theme 2)
**Research literacy of decision-makers**	Creation of confusion and doubt about policy decisions (Theme 2)	The extent to which decision-makers have the necessary skills and training to understand scientific methods	Assumption:<br>- Decision-makers who have the skills and training to understand scientific methods are able to make informed	Increase in *research literacy of decision-makers* leads to a decreased in:<br>- *The creation of confusion and doubt about policy decisions* (Theme 2)

		that underpin evidence-based policy recommendations.	policy decisions based on rigorous evidence.	
**Revolving doors**	Direct access to public sector decision-makers (Theme 1)	The extent to which revolving doors is prevalent. Revolving door "refers to the movement of individuals between positions of public office and jobs in the same sector in the private or voluntary sector, in either direction."[4]	Note:<br>- In some contexts, revolving doors can be seen a form of corruption, but in other contexts it can be a standard and accepted practice.	Increase in *revolving doors* enables or leads to increases in:<br>- *Direct access to public sector decision-makers* (Theme 1)<br>- *Government corruption* (Theme 1)<br>- *International business organisations favouring industry* (Theme 3)
**Shareholder primacy**	Corporate prioritisation of commercial profits and growth (Theme 3)	The extent to which corporations aims to maximise the value of a company for shareholders (i.e., profit maximisation) as a priority over other considerations, such as health, well-being, human rights, or the natural environment.	--	Increase in shareholder primacy leads to a decreased in:<br>- *Corporate prioritisation of commercial profits and growth* (Theme 3)
**Short-term view on policy decisions**	Corporate prioritisation of commercial profits and growth (Theme 3)	The extent to which politicians adopt a short-term view on policy decisions.	Assumption:<br>- Politicians may make decisions that lead to short term gain to stay in power. Such decisions may not necessarily lead to long term societal benefits.	Increase in *short-term view on policy decisions* enables or leads to an increase in:<br>- *Corporate prioritisation of commercial profits and growth* (Theme 3)<br>- *Government obliged to follow international trade and investment agreements* (Theme 4)
**Support for multistakeholder governance**	Industry leveraging policymaking, norms, rules, and processes (Theme 5)	The extent to which national and global governance institutions support the norm that multistakeholder.	Note:<br>- Multistakeholder governance is the inclusion of different stakeholders outside of government, such as the private sector or civil society to participate in policymaking, decision-making or policy implementation.<br>- There may be support for multistakeholder governance even though there may be a conflict of interest when making policy decisions with certain stakeholders.<br><br>Assumption:<br>- Industries leverage multistakeholder governance by arguing that	Increase in the *support for multistakeholder governance* enables or leads to an increase in:<br>- *Public-private partnerships* (Theme 1)<br>- *Direct access to public sector decision-makers* (Theme 1)<br>- *Industry leveraging policymaking, norms, rules, and processes* (Theme 5)

			governments ought to involve corporations in policy decisions (even if there may be conflicts of interest).<br><br>Examples:<br>- The UK's Responsibility Deal<br>- SDG 17 (Partnership for the goals): "Strengthen the means of implementation and revitalize the global partnership for sustainable development".	
**Tax avoidance and evasion**	Corporate prioritisation of commercial profits and growth (Theme 3)	The extent to which corporations exploit loopholes in the tax system to reduce tax liabilities (tax avoidance) and illegally attempt to evade the legal requirement to pay due taxes (tax evasion).	--	Increase in *tax avoidance and evasion* enables or leads to an increase in:<br>- *Corporate wealth* (Theme 3)<br><br>Increase in *tax avoidance and evasion* leads to a decrease in:<br>- *Government revenue* (Theme 3)
**Tax policy favouring corporations**	Corporate prioritisation of commercial profits and growth (Theme 3)	The extent to which different types of tax policies and laws - including income tax, corporate tax, capital gains, property tax, sales tax, excise taxes, and tax cuts - favour corporations.	Assumption:<br>- Generally, such tax laws would impact on UCI operations and the production and selling of their products, resulting in UCIs paying less tax.	Increase in *tax policy favouring corporations* leads to an increase in:<br>- *Corporate wealth* (Theme 3)<br>- *Pricing strategies* (Theme 3)<br>- *Externalities* (Theme 3)<br><br>Increase in *tax policy favouring corporations* leads to a decrease in:<br>- *Government revenue* (Theme 3)
**Ability of governments to hold corporations to account**	Industry leveraging legal and dispute settlement processes (Theme 4)	The extent to which government have the ability to investigate, prosecute and sanction problematic corporate behaviours in domestic jurisdictions and extra-territorial jurisdictions.	--	Increase in *ability of governments to hold corporations to account* leads to increases in:<br>- *Governance capability* (Theme 1)<br><br>Decrease in *ability of governments to hold corporations to account* leads to increases in:<br>- *Government corruption* (Theme 1)<br>- *Industry leveraging the legal and dispute settlement processes* (Theme 4)
**Shrinking role of the state**	Industry leveraging legal and dispute settlement processes (Theme 4)	The extent to which there is a "shrinking state", which refers to as the "retreat of the state from its customary intervention in regulating economic growth and promoting redistribution and the overall weakening of	Note:<br>- The shrinking state includes the "withdrawal of finance, services and staff as well as the failure to increase these resources to match growing needs, both of which are referred to as	Increase in the *shrinking role of the state* enables or leads to an increase in:<br>- *Government use of consultancy firms* (Theme 2)<br><br>*Support for multistakeholder governance* (Theme 1)

		the state as an institution in local/regional affairs"[5]	‘retrenchment’ or, in the European case, ‘austerity’.” [5]<br>- Due to funding cuts and retrenchments, government may lose the expertise and capability to conduct their own research. This may lead to the reliance on consultancies to conduct research to make policy recommendations.	Increase in the *shrinking role of the state* enables or leads to a decrease in:<br>- *Ability of governments to hold corporations to account* (Theme 4)<br>- *Governance capability* (Theme 1)

References

1. Gilmore AB, Fabbri A, Baum F, et al. Defining and conceptualising the commercial determinants of health. *The Lancet.* 2023;401(10383):1194-1213. doi:10.1016/S0140-6736(23)00013-2

2. Ulucanlar S, Lauber K, Fabbri A, et al. Corporate Political Activity: Taxonomies and Model of Corporate Influence on Public Policy. *International Journal of Health Policy and Management.* 2023;12(Issue 1):1-22. doi:10.34172/ijhpm.2023.7292

3. Legg T, Hatchard J, Gilmore AB. The Science for Profit Model—How and why corporations influence science and the use of science in policy and practice. *PLOS ONE.* 2021;16(6):e0253272. doi:10.1371/journal.pone.0253272

4. Transparency International. Revolving Door. n.d.; https://www.transparency.org/en/corruptionary/revolving-door. Accessed 24 November, 2022.

5. Lobao L, Gray M, Cox K, Kitson M. The shrinking state? Understanding the assault on the public sector. *Cambridge Journal of Regions, Economy and Society.* 2018;11(3):389-408. doi:10.1093/cjres/rsy026
